# RsmW, *Pseudomonas aeruginosa* small non-coding RsmA-binding RNA upregulated in biofilm versus planktonic growth conditions

**DOI:** 10.1186/s12866-016-0771-y

**Published:** 2016-07-19

**Authors:** Christine L. Miller, Manuel Romero, S. L. Rajasekhar Karna, Tsute Chen, Stephan Heeb, Kai P. Leung

**Affiliations:** Dental and Craniofacial Trauma Research and Tissue Regeneration Directorate, Institute of Surgical Research, JBSA Fort Sam Houston, TX USA; School of Life Sciences, Centre for Biomolecular Sciences, University Park, University of Nottingham, Nottingham, NG7 2RD UK; The Forsyth Institute, Cambridge, Massachusetts USA

## Abstract

**Background:**

Biofilm development, specifically the fundamentally adaptive switch from acute to chronic infection phenotypes, requires global regulators and small non-coding regulatory RNAs (sRNAs). This work utilized RNA-sequencing (RNA-seq) to detect sRNAs differentially expressed in *Pseudomonas aeruginosa* biofilm versus planktonic state.

**Results:**

A computational algorithm was devised to detect and categorize sRNAs into 5 types: intergenic, intragenic, 5′-UTR, 3′-UTR, and antisense. Here we report a novel RsmY/RsmZ-type sRNA, termed RsmW, in *P. aeruginosa* up-transcribed in biofilm versus planktonic growth. RNA-Seq, 5’-RACE and Mfold predictions suggest RsmW has a secondary structure with 3 of 7 GGA motifs located on outer stem loops. Northern blot revealed two RsmW binding bands of 400 and 120 bases, suggesting RsmW is derived from the 3’-UTR of the upstream hypothetical gene, PA4570. RsmW expression is elevated in late stationary versus logarithmic growth phase in PB minimal media, at higher temperatures (37 °C versus 28 °C), and in both *gacA* and *rhlR* transposon mutants versus wild-type. RsmW specifically binds to RsmA protein *in vitro* and restores biofilm production and reduces swarming in an *rsmY/rsmZ* double mutant. PA4570 weakly resembles an RsmA/RsmN homolog having 49 % and 51 % similarity, and 16 % and 17 % identity to RsmA and RsmN amino acid sequences, respectively. PA4570 was unable to restore biofilm and swarming phenotypes in Δ*rsmA* deficient strains.

**Conclusion:**

Collectively, our study reveals an interesting theme regarding another sRNA regulator of the Rsm system and further unravels the complexities regulating adaptive responses for *Pseudomonas* species.

**Electronic supplementary material:**

The online version of this article (doi:10.1186/s12866-016-0771-y) contains supplementary material, which is available to authorized users.

## Background

*Pseudomonas aeruginosa* is an opportunistic pathogen that thrives in a variety of environments. The ability of *P. aeruginosa* to adapt to different niches and establish both chronic and acute infections requires differential gene expression and phenotypic alterations ultimately coordinated by global regulators [1] and small non-coding regulatory RNAs (sRNAs) [2, 3]. The Csr/Rsm system is a regulatory network that is comprised of global RNA-binding regulators and sRNAs that regulate gene expression post-transcriptionally. The Csr/Rsm system, conserved in both Gram-negative and -positive bacteria, can impact both positively and negatively on the abundance of over 20 % of all mRNA, and controls a large variety of physiological processes (e.g. carbon metabolism, virulence, motility, quorum sensing, siderophore production, and stress response) [4–8].

RsmA, a member of the extensive family of CsrA homologs firstly described in *E. coli*, is an RNA-binding regulator that impacts the mRNA levels of 9 % of the genome of *Pseudomonas aeruginosa* [9]. Unlike other bacterial genera, *Pseudomonas* spp. have all been found to encode multiple RsmA homologs, including the redundant RsmE of *P. protegens* CHA0 and the RsmN paralogue of *P. aeruginosa* [10–12]. These homologs are directly regulated by RsmA, induced under various conditions, differ in sequence, secondary and tertiary structure, and have various RNA-binding affinities and specificities. Collectively, these RsmA homologs have overlapping and unique roles to fine-tune post-transcriptional gene regulation in *Pseudomonas*.

Generally, RsmA negatively regulates mRNA targets by binding to sites containing critical GGA motifs present in the 5’-untranslated region (5’-UTR) of the mRNA which impedes translation initiation or effects mRNA stability and turnover [13]. RsmA represses regulons necessary for establishing chronic infections including type VI secretion systems (T6SS), exopolysaccharide production, biofilm formation, and iron homeostasis [9, 14]. RsmA positively and indirectly regulates acute infection phenotypes through modulation of intracellular signaling networks (e.g. c-di-GMP levels), regulatory factors including genes associated with surface motility, type III secretion systems (T3SS), and type IV pili, as well as systems that operate through the cAMP/virulence factor regulator (Vfr) route [9, 14–19].

RsmA’s regulation, resulting in the switch from planktonic (acute) to biofilm (chronic) phenotypes, is ultimately cued by environmental signals recognized by three sensor kinases, GacS, RetS and LadS. RetS and LadS integrate these signals through repression or activation of the GacA/GacS two component regulatory system, respectively [15, 17, 20, 21]. The environmental signals that influence this pathway are still mostly unknown, however TCA cycle intermediates and temperature are thought to play a role [22, 23]. The Gac system antagonizes RsmA by inducing the transcription of redundant antagonist sRNAs, including RsmY and RsmZ in *P. aeruginosa* and RsmX, RsmY, and RsmZ, in *P. protegens* CHA0 and *P. syringae pv. tomato* DC300 [16, 24–28]. Interestingly, multiple homologous copies of RsmX exist in *P. syringae pv. tomato* DC300, *P. syringae* B728a, *P. syringae* 1448a, *P. mendocina* ymp, and *P. stutzeri* A1501 [28]. These small RNAs all have a secondary structure with numerous unpaired GGA motifs that act to sequester RsmA proteins from their targets [16, 26, 29].

The multiple small non-coding RNAs (RsmX, RsmY, and RsmZ) are thought to provide a dosage effect to help direct expression of specific RsmA/RsmN regulons. Even though these sRNAs are redundant, their transcriptions are, however, differentially regulated by a number of auxiliary factors which vary between them and between *Pseudomonas* species [10, 17, 27, 30, 31]. The architecture of the Rsm sRNA promoters is more complex than most bacterial promoters. Promoters of *rsmX, rsmY,* and *rsmZ* all contain an 18 bp upstream activating sequence (UAS) that is essential for their activation by the response regulator, GacA [21, 24, 27–29]. However, in the absence of GacA in *P. aeruginosa* transcription of *rsmY* and *rsmZ* is still achieved but to a lesser degree, suggesting the involvement of additional regulatory pathways [26]. In *P. aeruginosa,* MvaT and MvaU, global regulators and members of the histone-like nucleoid- structuring (H-NS) family of proteins, bind to an A + T rich region upstream of *rsmZ* to silence expression [25]. However, in *P. protegens* two recognition sites at the A + T region of the *rsmZ* promoter are bound by integration host factor (IHF); also a global regulator of the H-NS family. Due to the regulatory mechanisms of IHF, this suggests that DNA bending and temperature influence *rsmZ* transcription [22]. In *P. protegens* strains CHA0 and Pf-5, PsrA, a transcriptional activator of *rpoS* and repressor of fatty acid degradation, directly activates *rsmZ* expression [22, 32, 33].

Each Rsm sRNA is distinct, as demonstrated by differences in their temporal expression and mechanisms for turnover and stability. In *P. aeruginosa rsmY* transcription increases in parallel throughout cell growth, whereas *rsmZ* is induced sharply during the late exponential growth phase [26]. However, after 24 h of growth, RsmZ transcripts are degraded in *P. aeruginosa* [16] and interestingly, need to be eliminated before a biofilm can form. [30] Under biofilm growth conditions in *P. aeruginosa* RsmZ is degraded by CafA, a ribonuclease G activated by the two component system, BfiSR [30]. Expression of *rsmY* is negatively regulated through a phosphorelay event involving three sensor kinases (PA1611, PA1976, and PA2824) and HtpB (histidine-containing phosphorelay protein B) [31, 34]. RsmY is positively regulated by the sRNA chaperone, Hfq, which binds and stabilizes the RsmY transcript [35]. Taken together, there are both similar and unique mechanisms regulating these Rsm sRNAs.

Focusing on a specific Rsm sRNA and comparing it among different *Pseudomonas* species demonstrates similarities and differences in that sRNA’s expression patterns, regulators contributing to their transcription, stability/degradation mechanisms, and affinities for the different RsmA homologs. Regardless of the sRNA’s designation (X, Y, or Z), multiple Rsm sRNAs allow for the ability to steer, amplify, and/or fine-tune a response appropriate for each *Pseudomonas* species under different environmental niches.

This study demonstrates overlapping but unique aspects of a newly discovered RsmY/RsmZ-type of regulatory RNA analog in *P. aeruginosa*. Due to the unique sequence of this RNA having no homology with previously described Rsm regulatory RNAs, we have designated it RsmW.

## Results

### RsmW is upregulated under biofilm growth and appears to be processed out from upstream gene, PA4570

**Fig. 1:**
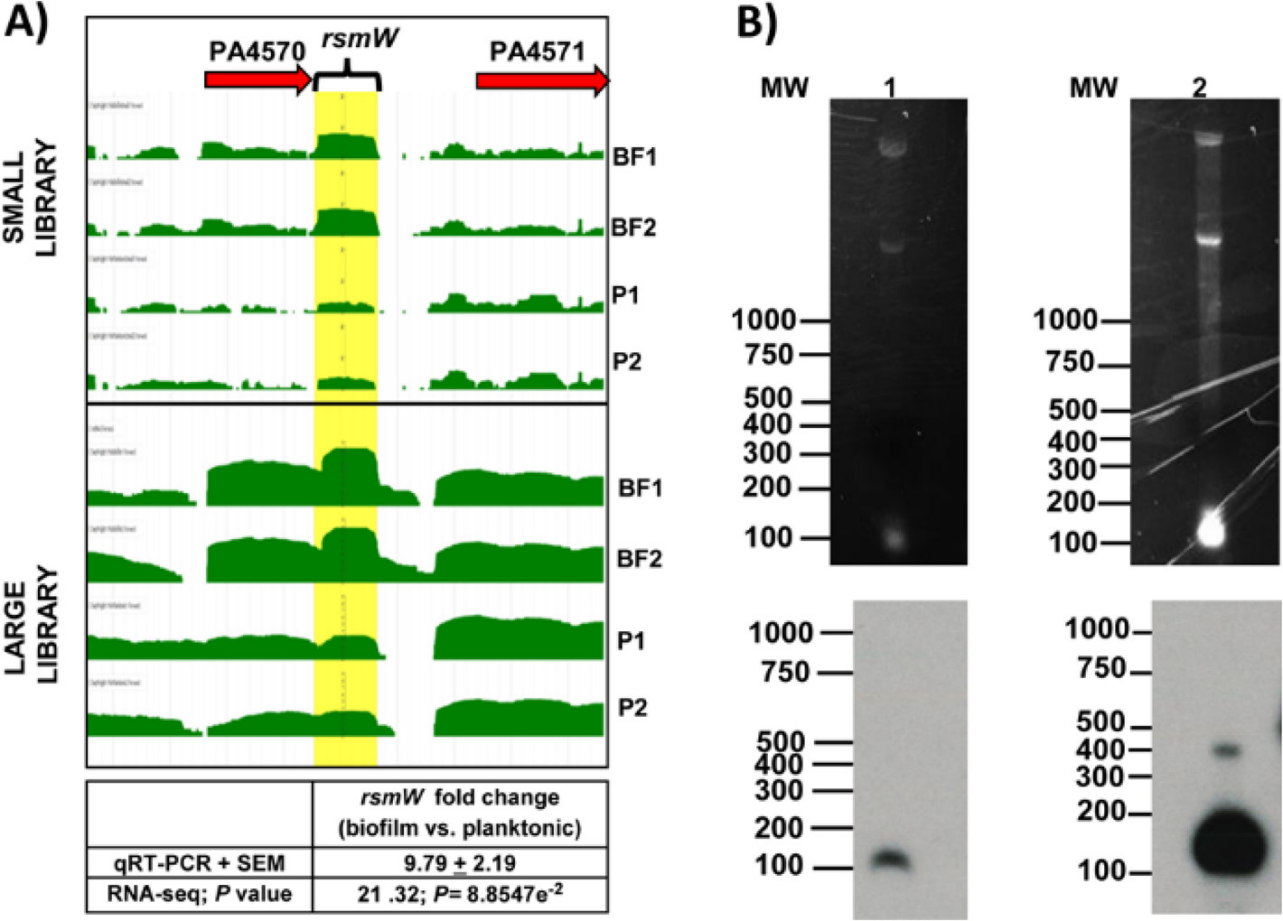
RsmW is upregulated under biofilm growth in *P. aeruginosa*. **a** RNA sequencing read mapping showing the genome region and levels of the predicted small RNA (RsmW) during biofilm (BF) and planktonic (P) growth states and from libraries prepared conventionally (LARGE Library) and adapted for small RNA retention (SMALL Library). RPKM (Reads Per Kilobase of transcript per Million mapped reads) were calculated for each small RNA to compare levels between conditions. RsmW was upregulated approximately 21- and 10-fold in biofilm versus planktonic conditions based on RNA-seq and qRT-PCR, respectively (bottom panel). RsmW was categorized as the 3’-untranslated region (3’-UTR) of gene PA4570 because it was less than 30 nucleotides apart from this open reading frame. **b** Northern blot analyses reveal two bands for RsmW. RNA (10 μg) from *P. aeruginosa* grown under planktonic or drip-flow biofilm conditions was run on denaturing glyoxal agarose gel (top panels), blotted, probed with radiolabeled riboprobes complementary to RsmW, and exposed for 1 hour (bottom panels). Hybridization bands of approximately 120 bases in planktonic and biofilm growth conditions and an additional band of approximately 400 bases in biofilm conditions are revealed. Lanes: MW, molecular weight ladder in number of bases; 1.) planktonic conditions; 2.) drip-flow biofilm conditions. Abbreviations: BF1, BF2: Biofilm replicates; P1, P2: Planktonic replicates; SEM: standard error of the mean

We sought to discover sRNAs important for biofilm growth in *P. aeruginosa*. Thus, samples grown for 24 h under drip-flow biofilm and planktonic growth conditions were harvested. Large (>200 bp) and small (<200 bp) RNA fractions were collected and analyzed using custom RNA sequencing (RNA-seq) to optimize for small RNAs. Gomez-Lozano et al. [36], who also performed RNA-seq of small RNA species in *P. aeruginosa,* detected and originally named RsmW as Pant420 (P. aeruginosa novel transcript 420) with 5118198-5118323 (126 bp) coordinates [36]. In our study, RsmW was upregulated approximately 21- and 10-fold in biofilm versus planktonic conditions based on RNA-seq and qRT-PCR, respectively (Fig. 1a). Similar to Ferrara et al. [37], a custom algorithm was used to categorize the detected small RNAs into intergenic, intragenic, 5′-UTR, 3′-UTR, and antisense sRNAs [37]. RsmW was categorized as a 3’-UTR of the 224 bp open reading frame (ORF) PA4570; however, the RNA-seq mapping profile demonstrated higher levels of RsmW compared to PA4570, suggesting either independent transcription or a processing event and higher stability of the *rsmW* RNA compared to the PA4570 mRNA.

To determine if RsmW was an independent transcript, we performed northern blot using a probe complementary to RsmW (Fig. 1b). A band approximately of 120 bases was revealed in both planktonic and biofilm growth conditions, however an additional band approximately 400 bases was present in biofilm growth conditions. These results suggest possible co-transcription of PA4570 and RsmW. Analysis using Ribosome Binding site calculator v 2.0 [38] indicates that *rsmW* RNA is unlikely to be translated because typical rates of translation were absent using all possible start codons.

### RsmW *in silico* analyses suggests its involvement in RsmA regulation

**Fig. 2:**
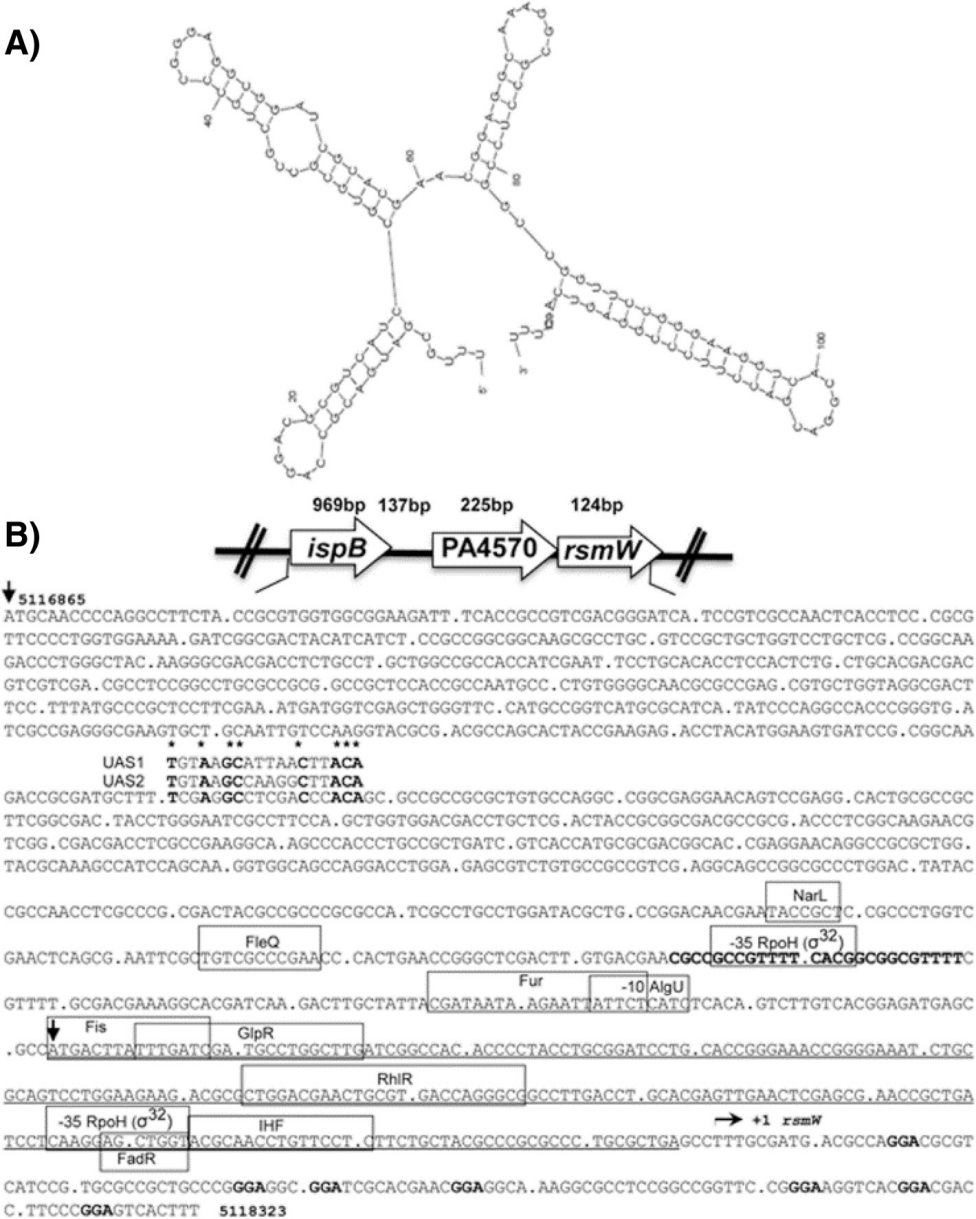
*In silico* analyses suggests RsmW involvement in RsmA regulation. **a** Secondary structure prediction of RsmW. Using Mfold and sequence based on 5’ RLM-RACE and RNA sequencing reveals that 3 out of 7 GGA motifs can be located on outer stem loops, patterns reminiscent of that of the small regulatory RNAs RsmX, RsmY and RsmZ, thus suggesting RsmW’s role in binding/sequestering the global regulator RsmA. Transcription of *rsmY* and *rsmZ* require GacA binding to upstream activating sequence (UAS; TGTAAG-N_6_-CTTACA). There is a weakly conserved GacA binding motif approximately 830 bp upstream of *rsmW*. **b** Promoter analysis of *rsmW*. Virtual Footprint/PRODORIC predictions (Munch et al. [40]) suggests binding sites and regulation of *rsmW* by RhlR, Fur, and IHF (shown in boxes). A conserved σ^70^ 35 site but not a -10 site appear upstream of *rsmW* within the PA4570 open reading frame (underlined). A -35 site 58 bp upstream of the 5’end of *rsmW* is predicted to overlap with a binding site for the heat shock sigma factor RpoH. Directly downstream of the -35 site is a putative IHF binding site, 46 bp upstream of the *rsmW* 5’end. The RLM-RACE predicted 5’end of RsmW is indicated with +1 and arrow. The first nucleotide of the start codons of the open reading frames are indicated with a downward arrow. Transcriptional terminator of *ispB* gene, upstream of PA4570, is indicated in bold. No transcriptional terminator can be predicted between PA4570 and *rsmW*

Using 5’ RLM-RACE and RNA-seq we determined the exact RsmW coordinates and predicted its secondary structure using Mfold [39]. The secondary structure highly resembles the small sRNAs, RsmZ and RsmY. RsmW contains 7 GGA motifs of which 3 are exposed in the single-stranded outer stem loops, suggesting its involvement and binding to RsmA (Fig. 2a). To further verify that RsmW is an RsmY/RsmZ-type of sRNA, we searched for potential binding sites for regulatory elements, such as GacA, a known activator of *rsmY* and *rsmZ*, using Virtual Footprint, an algorithm for regulon prediction in prokaryotes [40]. Transcriptional activation of *rsmY* and *rsmZ* by GacA requires a GacA binding site (upstream activating sequences UAS1 and UAS2; TGTAAG-N_6_-CTTACA). There is a weakly homologous GacA binding site approximately 830 bp upstream of RsmW (Fig. 2b). This is farther upstream than is the case for *rsmY* and *rsmZ* promoters, where the GacA sites are located -75 bp and -196 bp upstream, respectively [16, 26].

*In silico* promoter analyses suggests that under unique circumstances involving temperature and IHF binding protein, RsmW may be independently transcribed from PA4570 (Fig. 2b). A -35 site appears upstream of *rsmW* and within the PA4570 coding region. This -35 site is predicted to be overlapped by a binding site for RpoH, a heat shock sigma factor. Directly downstream of the -35 site is a putative IHF binding site. IHF has been shown to bind in response to temperature, binds to bent DNA, can create an open complex for RNA polymerase, and can promote transcription without the aid of other transcription factors [41–44]. On the other hand, the absence of any apparent transcription terminator following PA4570 supports co-transcription of *rsmW* and PA4570. Other potential regulatory elements of *rsmW* and/or PA4570 indicated *in silico* include RhlR (regulator of rhamnolipid biosynthesis and quorum sensing responses), Fur (ferric uptake regulator), AlgU (sigma factor and activator of alginate biosynthesis), FleQ (positive regulator of flagellar genes and mucin adhesion) and GlpR (repressor of glycerol uptake and metabolism) (Fig. 2b).

### RsmW levels increase in response to increasing temperature

Temperature is known to influence the expression of Rsm sRNAs in other pseudomonads [22, 29, 45, 46], therefore we wanted to determine the effect of temperature on RsmW levels. Using qRT-PCR we demonstrated that when *P. aeruginosa* PAO1 was grown at 37 °C versus 28 °C, RsmW levels increased approximately 5-fold, PA4570 levels increased 2-fold, and interestingly, RsmA levels decreased, although modestly, 1.5-fold (Additional file 1: Figure S1). Therefore, the heat responsive transcriptional regulatory elements (e.g. RpoH and IHF) seem likely contributors to the increased mRNA levels of RsmW at higher temperatures.

### RsmW expression is elevated in minimal medium, in stationary phase, and in both, *gacA* and *rhlR* mutants

**Fig. 3:**
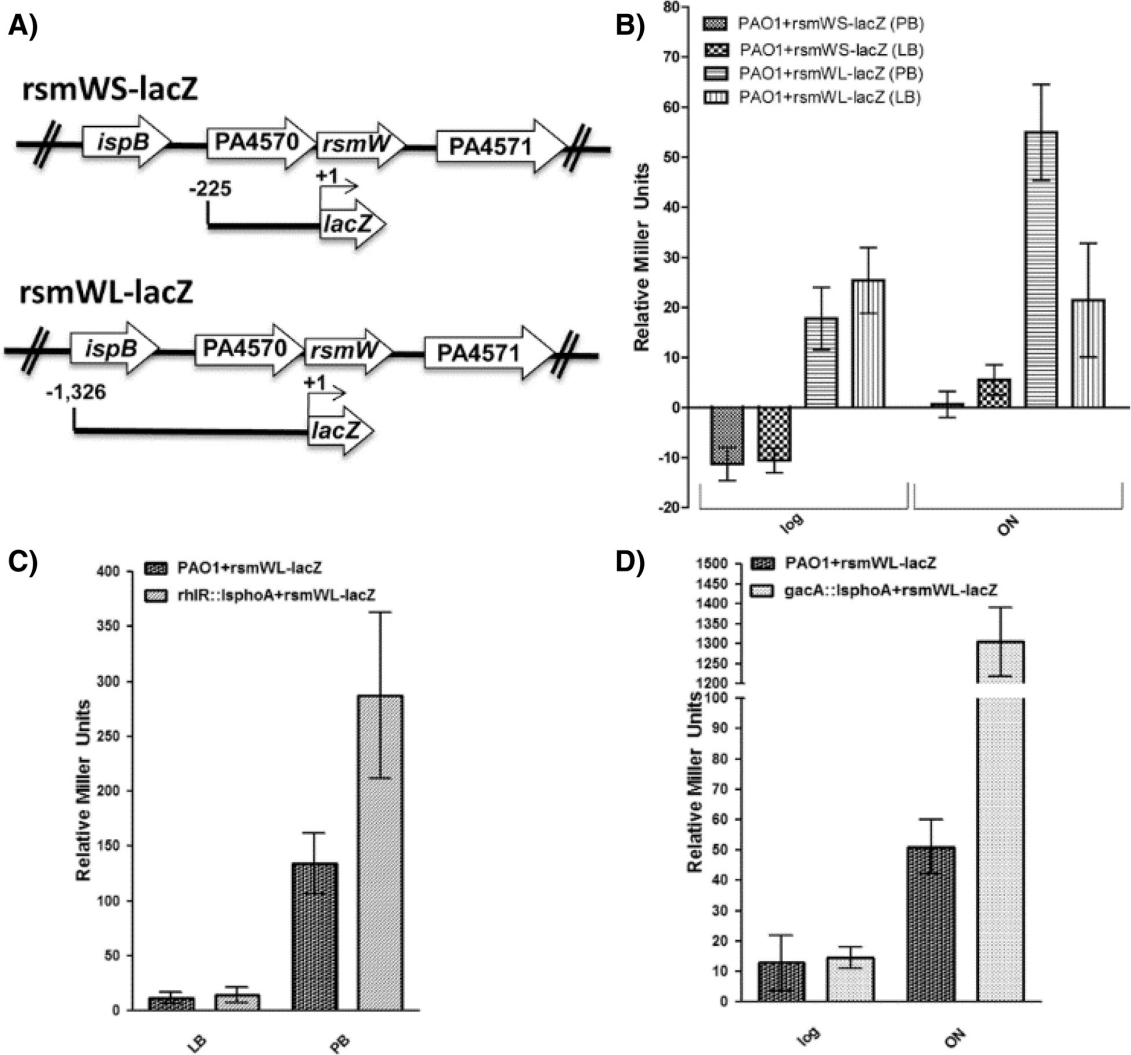
RsmW transcription is elevated in stationary phase and in *gacA* and *rhlR* deficient backgrounds. *P. aeruginosa* strains were grown in 5 ml cultures at 37 °C with 250 rpm shaking. Aliquots were processed and placed in a 96-well microtiter plate for absorbance readings at 420 nm as previously described by Griffith et al. [73]. The y-axis unit represents β-galactosidase activity normalized by OD600 nm of the cultures. To account for leaky expression or background noise arising from the strains or fusion system β-galactosidase activity from experimental strains was subtracted from a parental strain harboring an empty transcriptional fusion construct. All numbers indicate the average of three independent experiments. Error bars indicate mean ± standard error (s.e.m.). **a** Schematic representation of transcriptional fusions integrated into the chromosomes of wild-type and *rhlR* and *gacA* transposon mutants. **b** RsmW transcription is elevated in minimal media during stationary growth phase. *P. aeruginosa* strains were grown in minimal (PB) or nutrient rich (LB) media and samples at mid-logarithmic (OD600 nm = 0.6) and stationary phase (16 h) were analyzed for *lacZ* expression using both the rsmWS-lacZ and rsmWL-lacZ fusions. **c** RsmW transcription is elevated 2-fold in *rhlR* transposon mutant in minimal media during stationary growth phase. *P. aeruginosa rhlR* transposon mutants were grown in minimal (PB) or nutrient rich (LB) media and samples at stationary phase (16 h) were collected and analyzed using the rsmWL-lacZ fusion. **d** RsmW transcription is induced 27-fold in *gacA* transposon mutant. *P. aeruginosa gacA* transposon mutants grown in minimal (PB) media at mid-logarithmic (OD600 nm = 0.6) and stationary phase (16 h) were analyzed using the rsmWL-lacZ fusion. Abbreviations: log (mid-logarithmic phase); ON (stationary phase (16 hours))

Considering the regulatory sites predicted *in silico* upstream of *rsmW* and our observations that *rsmW* was upregulated under biofilm versus planktonic conditions, we identified other factors regulating *rsmW* transcription in PAO1 grown at both logarithmic (OD_600_ = 0.6) and late stationary (16 h) growth phases, and in minimal (PB) versus nutrient-rich (LB) media (Fig. 3). To further define the promoter requirements for RsmW, we assessed two chromosomally-integrated, transcriptional *lacZ* fusions. PAO1 + *rsmWS-lacZ* strain contained the first transcriptional fusion with a region consisting of the upstream gene, PA4570, 225 bp upstream of *rsmW*. A second transcriptional fusion consisting of 1,326 bp upstream of *rsmW*, including PA4570 and 1107 bp upstream of PA4570, was chromosomally integrated into PAO1 creating PAO1 + *rsmWL-lacZ* (Fig. 3a). Results were normalized by subtracting the OD_600_ of a control parental strain with an empty integrated construct (e.g. PAO1+ empty-*lacZ*). Our results demonstrated negligible β-galactosidase activity from the *rsmWS-lacZ* fusion, suggesting that this region was insufficient to drive *rsmW* transcription. However, the longer reporter fusion demonstrated that *rsmW* was up-transcribed approximately 3-fold more in late stationary versus logarithmic growth phase in PB minimal media (Fig. 3b). Furthermore, RsmW was upregulated approximately 2-fold more in PB late stationary cultures compared to LB late stationary cultures (Fig. 3b). However, there was no difference in reporter expression levels in LB media comparing logarithmic to late stationary cultures. All in all, production of RsmW is induced in minimal media after 16 h of growth (Fig. 3b).

As suggested by our *in silico* promoter prediction analysis for *rsmW*, RhlR may contribute to the regulation of *rsmW* expression. Therefore, the *rsmWL-lacZ* transcriptional fusion was chromosomally integrated into an *rhlR* transposon mutant (strain PW6883) creating *rhlR*::Is*phoA + rsmWL-lacZ*. Our results demonstrate that in the absence of RhlR in 16-h growth cultures, *rsmW* is upregulated approximately 2-fold, but only in PB minimal medium, not in LB, suggesting that RhlR may serve as a repressor of *rsmW* expression under minimal nutrient conditions (Fig. 3c).

Since *rsmY* and *rsmZ* are both transcriptionally activated by GacA we wanted to see if *rsmW* relied on GacA for transcriptional activation. The *gacA* transposon mutant (strain PW5341) with the *rsmWL-lacZ* transcriptional fusion demonstrated that *rsmW* transcription is increased 27-fold in the absence of GacA in PB minimal medium at 16 h, suggesting that the mechanism of induction occurs only after the culture has reached stationary phase (Fig. 3d). In contrast to RsmY and RsmZ in *P. aeruginosa* [26], GacA appears to directly or indirectly repress *rsmW* expression.

### RsmW can replace the functions of RsmY and RsmZ

We wanted to determine if RsmW could function in place of RsmY and RsmZ in *P. aeruginosa*. Therefore, we overexpressed RsmW in PASC659, strain deleted in both, *rsmY* and *rsmZ* genes, and assessed whether RsmW could restore the phenotypes of this ∆*rsmYZ* double mutant to wild-type levels. Using the 5’ end predicted by RLM-RACE, RsmW was overexpressed from a plasmid transcribing it from a constitutive *tac* promoter.

**Fig. 4:**
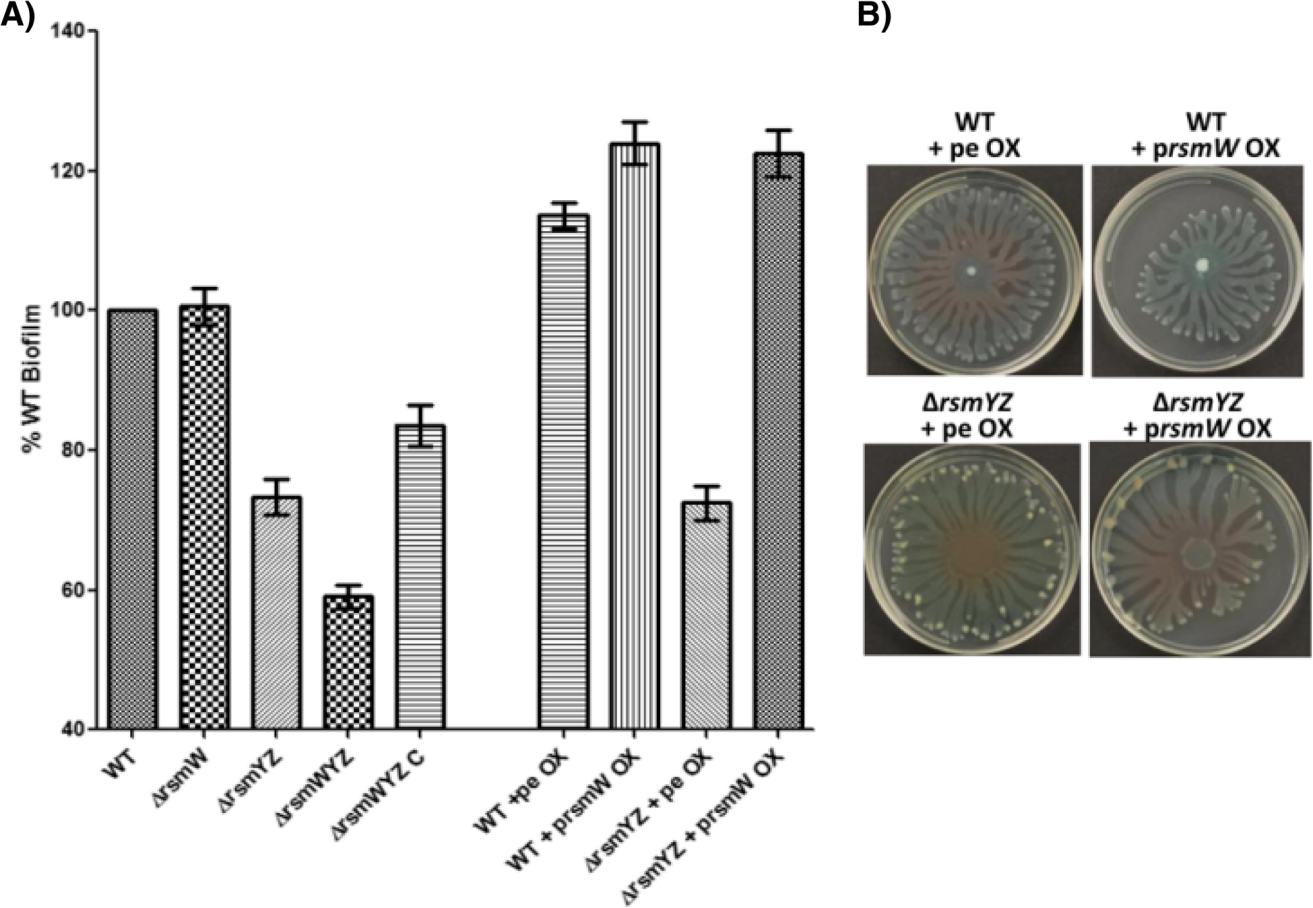
Complementation of ∆*rsmYZ* mutant by RsmW. **a** Overexpression of RsmW restores and increases biofilm production. Assessment of biofilms was performed using 0.1 % crystal violet staining method in 96-well microtiter plates after 24 hour of culture incubation at 37 °C in LB. An OD550 nm reading was taken and the average of 5 experiments is shown with standard error depicted. **b** RsmW expressed in a ∆r*smYZ* mutant background decreases swarming phenotype. Swarming motility assay demonstrated in *P. aeruginosa* strains after 24 hour incubation at 37 °C. The ∆*rsmYZ* mutant expressing RsmW from an overexpression plasmid (prsmW OX) has pigmentation colors more resembling wild-type compared to the ∆*rsmYZ* mutant carrying an empty plasmid. Strains: PAO1-N wild-type (WT); *rsmW*-deficient strain (∆rsmW); *rsmY*- and *rsmZ*-deficient strain (∆rsmYZ); *rsmW*-, *rsmY*- and *rsmZ*-deficient strain (∆rsmWYZ); ∆*rsmWYZ* strain with WT *rsmW* region swapped back in *cis* (∆rsmWYZ C); WT strain carrying empty overexpression plasmid (WT + pe OX); WT strain carrying plasmid overexpressing RmsW (WT + prsmW OX); ∆*rsmYZ* strain carrying empty overexpression plasmid (∆rsmYZ + pe OX); ∆*rsmYZ* strain carrying plasmid overexpressing RsmW (∆rsmYZ + prsmW OX)

The Δ*rsmYZ* double mutant produces less biofilm compared to the PAO1-N parental wild-type strain. Compared to the Δ*rsmYZ* mutant alone, the Δ*rsmWYZ* triple mutant was further impaired for biofilm production. This impairment was restored by complementing *rsmW* back in this strain in *cis* (strain Δ*rsmYZ* C) (Fig. 4a). The Δ*rsmYZ* double mutant overexpressing *rsmW* (strain Δ*rsmYZ* + p*rsmW* OX) demonstrated restored and increased biofilm levels compared to wild-type. Interestingly, overexpression of *rsmW* in the wild-type (strain WT + p*rsmW* OX) also increased biofilm production. Our results demonstrate that RsmW may compensate for the loss of RsmY and RsmZ and promote biofilm formation.

The Δ*rsmYZ* double mutant is a rapid swarmer compared to the wild-type strain, where all cells reach the edges of the Petri dish faster. However, the Δ*rsmYZ* mutant overexpressing *rsmW* demonstrated a reduction in swarming (Fig. 4b). Swarming differences between the Δ*rsmWYZ* and Δ*rsmYZ* mutant and between Δ*rsmW* and wild-type were not apparent in this assay (data not shown). Taken together, RsmW appears to partially complement for the loss of RsmY and RsmZ in regards to their contributions to swarming.

### RsmW binds RsmA *in vitro*

**Fig. 5:**
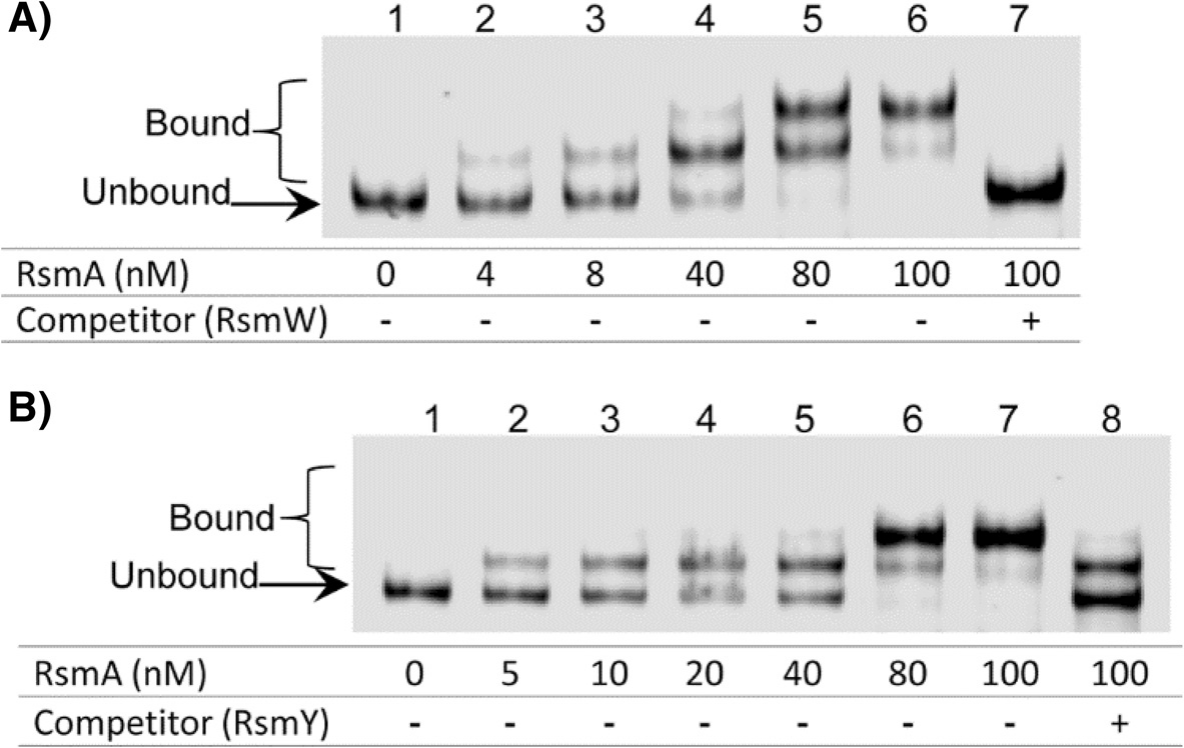
RsmA specifically binds to RsmW with high affinity. Electrophoresis mobility shift assay between RsmA and RsmW was carried out by synthesizing RsmW *in vitro* with T7 RNA polymerase. The fluorescently labeled RsmW (0.05 pmol in a final volume of 10 μl) was incubated in the absence (**a**, lane 1; **b**, lane 1) or presence of increasing concentrations of RsmA (**a**, lanes 2-7; **b** lanes 2-8), and in the presence of unlabeled specific competitor RNA, RsmW (5 pmol) (**a**, lane 7) or unlabeled competitor RNA, RsmY (5 pmol) (**b**, lane 8). From these results the dissociation constant (Kd) between RsmA and RsmW was determined to be 11.5± 1.5nM

RsmA binds RsmY and RsmZ at sites containing GGA motifs [13]. Due to the numerous GGA motifs present in RsmW we determined if RsmW could bind RsmA specifically and with high affinity. *In vitro* RNA binding assays were performed with recombinant RsmA and RsmW generated by *in vitro* transcription. Incubations with 0.05 pmol (5 nM) of RsmW with increasing concentrations of RsmA yielded one or two shifted bands demonstrating RsmW-RsmA complexes (Fig. 5a, lanes 2-6 and Fig. 5b, lanes 2-7). As has been suggested with RsmY [35, 47] and demonstrated with RsmZ [48], we speculate that the presence of multiple bands is the result of multiple RsmA proteins binding to the different sites containing the GGA motifs.

Competition assays were carried out and demonstrated that RsmW binds specifically to RsmA as addition of unlabeled RsmW resulted in a downshift (Fig. 5a, lane 7). Interestingly, addition of unlabeled RsmY competitor at the same concentration was only able to partially relieve the binding of RsmW with RsmA because two upshifted bands were still evident under these conditions (Fig. 5b, lane 8). Sonnleitner et al. [35] demonstrated a weaker binding affinity of RsmY for RsmA (Kd = 55± 7 nM) [35] than we observed with RsmW for RsmA (Kd = 11.5± 1.5nM).

Even though the experimental design between Sonnleitner et al. 35 and the present study have differences, taken together, RsmW appears to have higher affinity for RsmA than RsmY. RsmW has 7 GGA motifs like RsmY, but higher affinity could result from where the GGA motifs are localized in the secondary structure, or influences by neighboring secondary structures and nucleotides.

### RsmA regulates PA4570 and RsmW transcript levels and possible regulation of RsmW by Hfq

The lack of a transcriptional terminator between PA4570 and RsmW suggest that both co-transcribed. We hypothesize this co-transcript is bound by RsmA through the RsmW moiety, resulting in changes in the co-transcript’s stability or processing. To determine the contributions of RsmA on the transcript levels of PA4570 and RsmW, we utilized the conditional *rsmA* strain PASK10 [49], which is deficient of RsmA when grown in the absence of inducer and in which *rsmA* can be induced by the addition of IPTG. The RsmW and PA4570 mRNA levels were assessed by quantitative RT-PCR in PASK10 grown in LB with and without IPTG and at both mid-logarithmic and late stationary growth phases (16 h). PA4570 transcript levels increased 26-fold, whereas RsmW levels decreased 2-fold in PASK10 grown in the presence of IPTG compared to PASK10 grown without IPTG at mid-logarithmic growth phase (Additional file 2: Figure S2). There was no effect on PA4570 or *rsmW* RNA levels in cultures grown to late stationary phase (data not shown). These results demonstrate that in logarithmic growth RsmA increases PA4570 mRNA levels and decreases RsmW levels.

We hypothesize that RsmW may be stabilized and positively regulated by the small RNA chaperone Hfq, similar to RsmY. Therefore, RsmW RNA levels were assessed in an *hfQ*-deficient strain by qRT-PCR. Small regulatory RNA PrrF1 and RsmZ were used as a positive and negative control, respectively. RsmW levels decreased by a modest 2-fold in an *hfQ*-deficient strain compared to wild-type, suggesting a possible role of Hfq in stabilization of RsmW (Additional file 2: Figure S2).

### Characterization of PA4570 and similarities to RsmN and RsmA

**Fig. 6:**
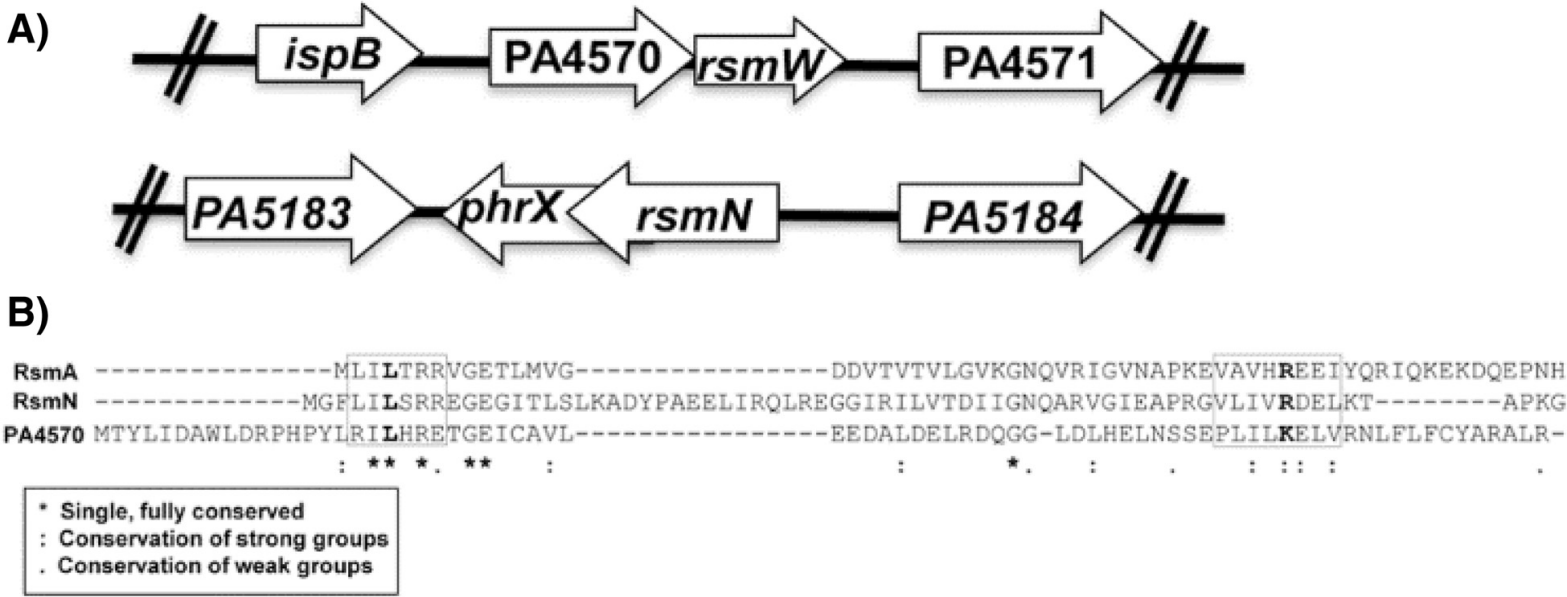
PA4570, upstream of *rsmW*, resembles an RsmA/RsmN homolog. **a** Similarities in gene topology between RsmN and PA4570 upstream of RsmW. **b** Primary sequence alignment of *P. aeruginosa* RsmA, RsmN, and PA4570 demonstrating sequence similarities. RsmN and RsmA has 64 % similarity and 31 % identity in sequence. RsmN and PA4570 has 51 % similarity and 17 % identity in sequence. RsmA and PA4570 has 49 % similarity and 16.3 % identity in sequence. Conservation stringency of residues is depicted using symbols below. (*) Single, fully conserved residue; (:) Conservation of strong groups; (.) Conservation of weak groups. L4 and R44 residues important for RsmE binding to *hcnA* 5’-UTR are fully and strongly conserved in PA4570, respectively, and indicated in bold. Boxes indicate region 1 and region 2 of CsrA, RsmA, and RsmE homologs thought to mediate RNA binding

Due to a possible linkage of PA4570 and RsmW, we characterized PA4570. Interestingly, sequence and genomic topology similarities between RsmN, RsmA, and PA4570 suggest that PA4570 might be another RsmA/N homolog (Fig. 6a, b). Specifically, sequence alignment demonstrated that PA4570 has 17 % identity and 51 % similarity to RsmN and 16 % identity and 49 % similarity to RsmA. PA4570 is predicted to translate into a protein of 74 amino acids, similar to RsmN (71 amino acids) and RsmA (61 amino acids). Also, PA4570 has many basic residues (10/74) similar to RsmA (9/61) and RsmN (11/71). PA4570’s basic residues and region of highest conservation are within the two regions known to be involved in RNA binding by the Csr/Rsm homolog proteins (Fig. 6b). L4 and R44 residues are important for RsmE binding to the *hcnA* 5′-UTR [50]. In PA4570, L4 is conserved, but R44 is replaced with a conservative substitution of a lysine (K) (Fig. 6b).

Due to this sequence homology, we wanted to see if PA4570 could complement for RsmA, thus PA4570 or PA4570 together with *rsmW* were overexpressed in a *P. aeruginosa* ∆*rsmA* mutant strain*.* The ∆*rsmA* mutant forms robust biofilms and is defective for swarming. Overexpressing both PA4570 with *rsmW* or PA4570 alone could not restore the ∆*rsmA* mutant to a wild-type phenotype (Additional file 3: Figure S3A and Additional file 3: Figure S3B). We also wanted to look if PA4750 could complement CsrA, the RsmA homolog of *E. coli* that inhibits glycogen synthesis [51]. Heterologous overexpression of PA4570 in a wild-type *E. coli* had no effect on glycogen production, as indicated by no apparent change in colony streak color after iodine staining (Additional file 3: Figure S3C). Interestingly, *E. coli* heterologously overexpressing PA4570 with *rsmW* or *rsmW* alone showed an increase in glycogen accumulation and supports that RsmW can serve as a sRNA antagonist of *E. coli’s* CsrA.

Our data suggests that although PA4570 may show sequence homology to RsmA/N homologs, it does not appear to be a functional equivalent to these homologs.

## Discussion

### RsmW

Many *Pseudomonas* species harbor three types of Rsm riboregulators (RsmX, RsmY, RsmZ), however until now *P. aeruginosa* has been shown to have only RsmY and RsmZ. Interestingly, some *Pseudomonas* species can carry up to five RsmX homologs [28]. The reason behind having so many Rsm riboregulators is still unclear but suggests their importance for increasing the dynamic nature and robustness of the Rsm regulatory network and for providing specificity and phenotypic diversity required for the various *Pseudomonas* species and their unique niches. We disclose RsmW, another Rsm sRNA, but which is unique in many ways.

Many studies have shown that 3’-UTRs serve as genomic reservoirs for regulatory sRNAs [52], and unlike RsmZ and RsmY which are independently transcribed from promoter elements, RsmW appears to be processed out from the 3’-UTR of PA4570, a hypothetical ORF of 224 bp. Aside from the -35 and -10 site upstream of PA4570, a -35 site 58 bp upstream of *rsmW* was predicted *in silico.* However, lack of an apparent -10 site, absence of a terminator between PA4570 and *rsmW*, and our transcription studies which demonstrated that *rsmW* could not be expressed independently of the upstream gene promoter elements, suggests that PA4570 and *rsmW* make up one transcriptional unit.

Compared to RsmY and RsmZ, production of RsmW is induced under different conditions. Our work and Wurtzel *et al.* [53] demonstrate that higher temperatures (37 °C versus 28 °C) positively affect the expression and levels of RsmW, but in contrast higher temperatures had much less of an effect on *rsmY* and *rsmZ* expression. Interestingly, the aforementioned -35 site, 58 bp upstream of *rsmW,* is predicted to be bound by the heat responsive sigma factor, RpoH. Immediately downstream of this -35 site is a predicted IHF binding site. IHF also binds to DNA in response to temperature [44]. Many studies show that temperature regulates Rsm sRNA expression [22, 29, 45, 46]. The fact that RsmW levels are increased at higher temperatures and *rsmW* is conserved in the opportunistic human pathogen *P. aeruginosa* and not in the other pseudomonads provides a unique mechanism for fine-tuning the Rsm regulatory circuit specific for *P. aeruginosa*.

RsmW expression patterns vary from those of *rsmZ* in *P. aeruginosa.* We showed that RsmW is upregulated in stationary phase growth and 24 h biofilms compared to mid-logarithmic growth phase and that overexpression of *rsmW* enhances biofilm development. In contrast, RsmZ RNA is absent from stationary phase cells after 24 h [16]. Studies also show that biofilm development requires reduced levels of RsmZ, but not RsmY; and overexpression of *rsmZ* is sufficient to arrest biofilm formation [30]. Overall, our studies suggest that enhancement of biofilm formation by RsmW is due to its direct interactions with RsmA.

Levels of RsmW are regulated differently from RsmY and RsmZ because *rsmW* is not transcriptionally activated by GacA. Based on our transcriptional reporter studies, GacA appears to have a negative effect on *rsmW* transcription, demonstrating another scenario where RsmW can be induced under conditions unique from RsmY and RsmZ. In *Yersinia pseudotuberculosis,* the GacA/GacS system (BarA/UvrY) activates transcription of only one of the two CsrA antagonist sRNAs, CsrB [54]. Expression of the second sRNA, CsrC, is activated by the PhoQ/PhoP two component system [55]. An RhlR binding site predicted *in silico* upstream of *rsmW* suggested a possible activational mechanism for *rsmW*. However, our transcriptional studies suggest that RhlR represses *rsmW* expression because *rsmW* expression is upregulated in an *rhlR* transposon mutant in late stationary phase in minimal media. Nevertheless, RhlR regulation is dynamic and RhlR can serve as both an activator [56, 57] and a repressor [58, 59]. RhlR expression is upregulated under late stationary growth phases [60], in nitrogen and phosphate limiting conditions [59, 61], and in mature 3-day-old biofilms [62]; a pattern reminiscent of *rsmW* expression. Therefore, RhlR regulation of RsmW may be multifactorial and induction of *rsmW* transcription by RhlR may occur under conditions not tested in our study. Collectively, the unique expression patterns and regulation of RsmW implies a specific role for RsmW in the RsmA/RsmN regulatory network.

### PA4570

Previous transcriptional studies demonstrated that the regulon of RsmA in *Pseudomonas* spp. is smaller than expected when comparing to the CsrA regulon in other bacteria [9]. A recent discovery that *Pseudomonas* harbors another RsmA homolog, RsmN, helped explain this by expanding the number of targets controlled by the system [11, 12]. Even though PA4570 was unable to complement for RsmA or CsrA in terms of glycogen metabolism, swarming, and biofilm production (Additional file 3: Figure S3), this protein may still be a distant homolog of RsmA or RsmN. PA4570 may have been horizontally acquired or come from a gene duplication of RsmA or RsmN and over time acquired mutations making it dysfunctional or highly specialized. Feasibly, our experiments may not be suitable to recapitulate the conditions necessary to reveal PA4570’s function in the RsmA/RsmN regulon.

Nevertheless, PA4570’s linkage to *rsmW* and *in silico* similarities to RsmA and RsmN provides thought-inducing evidence of its role as an RsmA/RsmN homolog. PA4570 shares a sequence similarity with the homologs comparable to what RsmA shares with RsmN, and it maintains the conservation with the homologs across the RNA-binding region and with known critical residues.

In *Pseudomonas* spp. the Rsm system functions with many RsmA homologs, comprised of various affinities for both their targets and sRNA inhibitors, and coordinates events as a result of stoichiometric shifts. If PA4570 is an RsmA/RsmN homolog we propose a model where PA4570 and RsmW are linked to help regulate the stoichiometric shift and possibly expand the regulon (Additional file 4: Figure S4). Interestingly, the regulatory linkage between PA4570 and RsmW can be examined by assessing our RNA-seq study (Additional file 5: Table S1). RNA sequencing results of the Δ*rsmW* mutant compared to wild-type demonstrated that PA4570 was also down-regulated approximately 3-fold. It is possible that deleting the 3’-UTR of PA4570 may affect the overall transcript stability making it difficult to determine if the changes in gene expression are due to PA4570 or RsmW. However, we do not believe that it is mere coincidence that many of the genes differentially expressed are part of the RsmA regulon and were expressed in a pattern indicative of an alleviation of repression of RsmA presumably by the absence of RsmW. So on the other hand, PA4570 may have no function other than to regulate RsmW production, where RsmW activation and maturation occurs after it is processed out from the PA4570-*rsmW* transcript, a mechanism similar to the recently discovered nitrogen responsive sRNA, NrsZ, in *P. aeruginosa* [63].

## Conclusion

In conclusion, RsmW is a Rsm sRNA that is upregulated in *P. aeruginosa* grown in nutrient-limiting conditions, biofilms, and at higher temperatures. Unlike *rsmY* and *rsmZ*, *rsmW* is not transcriptionally activated by GacA and RsmW appears to be processed out from the 3’-UTR of PA4570. Our study is the first characterization of the hypothetical ORF, PA4570, and further unravels the complexities of the global Gac/Rsm system that provides adaptive post-transcriptional modulations of gene expression in *Pseudomonas* species.

## Methods

### Bacterial strains and growth conditions

Details regarding the source of the strains including *Pseudomonas aeruginosa* wild-type (PAO1, Nottingham subline), and its derived Δ*rsmA* mutant (PAZH13), inducible *rsmA* (PASK10) strains are described in Additional file 6: Table S2. *P. aeruginosa* strains were routinely grown in LB at 37 °C. Concentrations of antibiotics used for *E. coli* were: kanamycin 50 μg ml^-1^, 100 μg ml^-1^ ampicillin or 50 μg ml^-1^ carbenicillin, 10 μg ml^-1^ gentamicin, 10 μg ml^-1^ tetracycline, and 100 μg ml^-1^ spectinomycin. For *P. aeruginosa*: 200 μg ml^-1^ carbenicillin, 50 μg ml^-1^ gentamicin, 100 μg ml^-1^ tetracycline, and 200 μg ml^-1^ streptomycin were used. Strain PASK10 was grown to an OD_600_ of 0.5 either in the absence (uninduced) or in the presence (induced) of Isopropyl β-D-1-thiogalactopyranoside (IPTG) at a final concentration of 1 mM. The cultures were collected during stationary growth phase (16 h after inoculation) and assayed in triplicate.

For small RNA detection, RNA-seq samples from mid-logarithmic (OD_600_ = 0.6) planktonic and 24-h drip-flow biofilm cultures were harvested. Cultures were grown overnight in tryptic soy broth (TSB), next day 250 μL of culture was seeded into 5 mL of 20 % Brain Heart Infusion (BHI++) (7.4 g/L BHI, 4 g NaCl, 2 g/L glucose) and allowed to reach an OD_600_ of 0.5. Cultures were diluted to an OD_600_ = 0.05 in phosphate-buffered saline (PBS) and allowed to incubate in the drip flow apparatus for 2 h to promote attachment before BHI media was supplied. For RNA-seq samples of Δ*rsmW* mutant and wild-type strains (Additional file 5: Table S1), bacteria were grown overnight in Peptone Broth (PB) medium [64], diluted by 5 % into fresh PB medium, allowed to reach on OD_600_ of 0.5, after which the cultures were re-diluted to an OD_600_ = 0.05 and grown to late stationary growth phase (16 h).

### Strain construction

#### Deletion mutants and WT “gene-swap” strains

For WT “gene-swap,” the wild-type genes were restored in the *ΔrsmW*, *Δ4570/ΔrsmW* and *Δ4570* strains by recombining the wild-type gene in the same place as the mutation as previously described [65]. In-Fusion HD Cloning Kit (Clontech Laboratories) was used following manufacturers suggestions to design primers (Additional file 7: Table S3) and construct plasmids for allele replacement. 1 kb upstream and downstream regions of the targeted gene were amplified by PCR from *P. aeruginosa* chromosome. For deletion constructs, a ~1 kb gentamycin antibiotic cassette, pucGM, was PCR-amplified from pJQ200. For WT “gene-swap” constructs, a ~1 kb streptomycin antibiotic cassette, aadA, was PCR-amplified from pCR2.1-P_flgB_-aadA. The three fragments for the deletion and WT “gene-swap” constructs were purified by gel electrophoresis and incubated with the HD-Infusion enzyme. Nested PCR of HD-Infusion reaction mixture was carried out using LA Taq polymerase (TAKARA BIO INC.) and the resulting 3-kb product was cloned into pCR2.1 TOPO-TA linear vector (Invitrogen). The deletion and WT “gene-swap” vectors were finally linearized using XbaI and SacI and electroporated into *P. aeruginosa* to achieve allelic replacement as described previously [66].

#### RsmW, PA4570 overexpression strains

567-bp and 242-bp DNA fragments of PA4570-*rsmW* or PA4570 alone were amplified by PCR, digested with SacI and XbaI and cloned into pJAK12 digested with the same enzymes. To transcribe *rsmW* from the +1 nucleotide and to remove the ribosome binding site from the pJAK12 expression vector, first site-directed mutagenesis was performed using *PfuTurbo* DNA polymerase (Agilent) to engineer an EcoRI site upstream of the tac promoter (ptac). Subsequent EcoRI digest resulted in removal of the tac promoter from pJAK12. A 146 bp *rsmW* fragment was PCR-amplified using a forward primer containing the ptac and starting at the +1 transcriptional start site determined by 5’ RLM-RACE, digested with EcoRI and SalI, and ligated into pJAK-ptac plasmid digested with the same enzymes. Resulting p*4570-rsmW*, p*4570*, and p*rsmW* OX plasmids as well as the empty vector pJAK12, were electroporated into *P. aeruginosa* as described previously [66].

#### Transcriptional reporter fusions

Two regions upstream of *rsmW*, 365-bp and 1,114-bp long, were amplified by PCR using oligonucleotides listed in Additional file 7: Table S3. PCR products were digested with EcoRI/BamHI, and ligated into mini-CTX *lacZ* for transcriptional fusion constructs. For transcriptional fusions constructs for the *rhlR* (strain PW6883) and *gacA* (strain PW5341) transposon mutants the tetracycline antibiotic cassette was replaced with a gentamycin cassette using AclI. The resulting plasmids were electroporated into *P. aeruginosa* strains as described previously [66]. The constructs were integrated into the attB site and the antibiotic resistance marker was removed using pFLP2 as described previously [67].

### RNA-sequencing

RNA sequencing was performed by SeqWright (Houston, TX) and the custom strand-specific sequencing libraries, specifically enriched for small RNAs (<200 bp), were generated as previously described [36]. Briefly, 2 to 5 μg total RNA was used for preparation of both (large and small RNA) strand-specific RNA-seq libraries. For large and small RNA libraries, rRNA, including 5S rRNA, was depleted from total RNA using the Ribo-Zero Magnetic kit (Epicentre). The directional RNA-Seq libraries for large and small RNA were developed using the NEXTflex directional RNA-Seq (dUTP-based) kit (Bioo Scientific). For small RNA libraries specifically, ethanol precipitation was used for cleanup steps to promote small RNA retention. Depleted RNA from small RNA samples were treated with Tobacco Acid Pyrophosphatase (Epicentre) at 37 °C for 60 min to promote correct adapter ligation followed by organic extraction cleanup (with 25:24:1 phenol:chloroform:isoamyl alcohol) and ethanol precipitation of RNA. Small RNA libraries were prepared using the TruSeq Small RNA sample preparation kit for Adapter ligation (Illumina) and sequenced using a paired-end protocol and read lengths of 100 nucleotides. Both small and large RNA-Seq libraries were subjected to the quantification process and pooled for cBot amplification and a subsequent sequencing run with a HiSeq 2000 platform (Illumina).

After the sequencing run, de-multiplexing with CASAVA was employed to generate a FASTQ file for each sample. Single-end nucleotide reads were mapped to the annotated draft genomic sequence of *P. aeruginosa* PAO1-UW (GenBank accession no. NC_002516.2) using the software Bowtie [68]. The mapped reads were separated into the forward and reverse complement directions using the “mpileup” option of the SAMtools software [69]. The mapped reads on each strand were visualized in the JBrowse genome viewer [70] for sequencing quality.

### Small RNA detection and categorization

A custom computer script (Miller et al., manuscript in preparation) was developed to detect and categorize sRNAs based on the single nucleotide RNA read-count profiles. The detected sRNAs were then categorized into the following 5 classes: Class I - intergenic, Class II5 - 5’-UTR, Class II3 - 3’-UTR, Class III - antisense, and Class IV - intragenic based on the criteria published by Ferrara et al. [37].

### Differential expression analyses

For differential transcript level analysis of genes and small RNAs, raw read counts for the *P. aeruginosa* transcripts were determined with a Perl script based on the mapped read profiles determined above. The read counts were subjected to the Bioconductor software package “DESeq” [71] to evaluate the differential levels for the RNAs between experiments. Two sequencing runs derived from two independently conducted experiments were used in the DESeq analysis.

The RNA-seq sequence data comparing WT and the *ΔrsmW* deletion mutant were deposited to the NCBI Sequence Read Archive under the BioProject accession number PRJNA326119. The dSample_WT corresponds to wild-type *Pseudomonas aeruginosa*, the university subline, PAO1-UW. The dSample_MT corresponds to the *ΔrsmW* deletion mutant that was generated by replacing the region between coordinates 5118198-5118322 with a ~1 kb gentamycin antibiotic cassette, pucGM, derived from plasmid pJQ200.

### RNA extraction and quantitative RT-PCR

RNAprotect (Qiagen) was added immediately to bacteria samples for RNA harvesting. RNA was extracted using mirVana miRNA Isolation Kit for whole RNA. Genomic DNA was removed by treatment with DNAse I (Ambion). RNA was quantified using a Nanodrop spectrophotometer (Invitrogen) and reverse transcribed to cDNA using the iScript Select cDNA synthesis kit (Bio-Rad). The absence of DNA contamination was confirmed using a minus-reverse transcriptase (“-RT”) control demonstrating a CT value 10 cycles higher than the reverse transcribed samples. Quantitative real-time PCRs were performed using SYBR green master mix (Bio-Rad) with specified primers (Additional file 7: Table S3) and analysis by the ABI Prism 7300 system (Applied Biosystems) with relative changes, using *fabD* and 16S housekeeping genes, and fold difference with 2-ΔΔCt method. Unpaired student’s *t*-test and *P* < 0.05 (Prism) were implemented.

### Northern

NorthernMAX-Gly system Kit (Ambion) was used for 1 % agarose gel and running buffer. The samples were denatured for 30 min at 50 °C in an equal volume of glyoxal load dye. Nucleic acids were transferred to a GE/Whatman Nytran SuPerCharge 0.45um 11 × 14 cm membrane using 20× Saline Sodium Citrate (SSC) and a TurboBlotter apparatus for 16 h. Prehybridization (1 h at 68 °C) and hybridization (16 h at 68 °C) was carried out in ULTRAhyb buffer (Ambion) at a volume of 10 ml per 100 cm^2^ following NorthernMAX Gly kit instructions. The probe was generated using T7 RNA polymerase (Fermentas) and ^32^P CTP (Perkin Elmer), before being purified on G50 Sephadex columns. The probe was added to the hybridization buffer at approximately 1.5 × 10^6^ dpm/ml. After hybridization, the membranes were washed using Ambion’s low stringency wash solution #1 (20 ml per 100 cm^2^) at room temperature for 20 min. with shaking. The high stringency wash was performed twice for 20 min. each wash at 68 °C using 20 ml per 100 cm^2^. Finally, membranes were exposed for varying times to Kodak Biomax MS films with an intensifier screen at −70 °C.

### 5’ RLM-RACE

Mapping of the 5’ transcriptional starting nucleotide was performed using First Choice RLM-RACE Kit (Ambion) per the manufacturer’s recommendations. Briefly, 10 μg of DNAse-treated drip-flow RNA was treated with tobacco alkaline pyrophosphatase for 1 h at 37 °C and adapters were ligated before RNA was reverse transcribed using M-MLV RT. Nested PCR using LA Taq polymerase (TAKARA BIO INC.) was carried out using serial dilutions of the reaction mixture. The PCR products were separated by agarose gel electrophoresis, DNA bands were eluted and cloned into pCR2.1 TOPO vector (Invitrogen), and the inserts were sequenced using the M13R primer.

### β-galactosidase assays

β-galactosidase activities were determined by the Miller method [72] using the β-galactosidase Assay Kit (Genlantis) with the addition of 0.1 % sodium dodecyl sulfate and 0.27 % 2-mercaptoethanol to the lysis buffer. *P. aeruginosa* strains were grown in 5 ml cultures at 37 °C with 250 rpm shaking overnight. Next day, 100 μL of culture was seeded into 5 ml of media and allowed to reach an OD_600_ of 0.5. Cultures were diluted to an OD_600_ of 0.05 and samples were collected at mid-logarithmic (OD_600_ 0.6) and stationary phase (16 h after inoculation) for β-galactosidase activity. A 96-well microtiter plate was used as previously described [73]. All numbers indicate the average of three independent experiments with standard error.

### Swarming assays

Swarming motility assays were performed on plates containing 0.5 % w/v bacto agar (Difco), 8 g of nutrient broth (Oxoid) l^-1^, and 0.5 % w/v D-glucose as previously described [16, 74]. Bacteria were grown in Luria-Bertani lysogeny broth (LB) medium overnight and 3 μl of culture was spotted in four independent replicates. Swarming was observed after 24 h of incubation at 37 °C.

### Glycogen assays

Function of PA4570, PA4570-*rsmW*, or *rsmW* in the Csr system of *E. coli* was assessed by staining glycogen with iodine as previously described [51]. Briefly, cells were streaked on Kornberg medium (1.1 % K_2_HPO_4_, 0.85 % KH_2_PO_4_, 0.6 % yeast extract, and 1.5 % glucose) plates containing 1 mM IPTG. Plates were exposed to vapors from iodine solution (0.01 M I_2_ and 0.03 M KI).

### Biofilm assays

Assessment of biofilms was performed using the crystal violet method and performed in 96-well microtiter plates. Strains were inoculated into 200 μl of LB medium, and after 24 h of incubation at 37 °C the growth medium was removed, the wells stained with 0.1 % crystal violet, the biofilms dissolved with 33 % acetic acid, and the OD_550_ nm reading taken.

### Protein production and RNA preparation

The pET-28b(+) expression system (Novagen) was used to produce His-tagged RsmA (His6-Thb-RsmA) within host *E. coli* C41(DE3) cells [12]. Overnight culture (10 ml) of C41 (DE3) harboring the expression plasmid was used to inoculate LB rich medium (1 L) containing the appropriate antibiotic. This cell culture was incubated with shaking (37 °C, 200 rpm) until the OD_600_ was 0.6-0.9 (~3 h), at which point production of His6-Thb-RsmA was induced by the addition of IPTG to a final concentration of 0.3 mM. The induced cell culture was incubated overnight with shaking (30 °C, 200 rpm, ~16 h), at which point OD_600_ reached ≥1.6. The cells were harvested by centrifugation and the cell pellet was stored at −80 °C until required. His6-fusion protein was purified by using Ni-NTA Fast Start Kit (Qiagen) following manufacturer’s procedure.

### Gel mobility shift assays

DNA template corresponding to *rsmW* was amplified by PCR using primers that incorporated a T7 promoter at the 5’ end and a 17 nt extension at the 3’ end. The PCR product was then used for RNA synthesis *in vitro* using the MAXIscript T7 kit (Life Technologies). The RNA obtained was visualized using the method described in Ying et al. [75] consisting of hybridization of an ATTO700-labeled DNA primer to the 3’ extension of the RNA [75], adjusting the fluorescent primer concentration to a 20-fold excess with respect to the RNA concentration in order to maximize hybridization and detection. The indicated concentrations of His6-Thb-RsmA were incubated with RsmW RNA (0.05 pmol) in 1× binding buffer (10 mM Tris-Cl pH 7.5, 10 mM MgCl_2_, 100 mM KCl), 0.5 μg/μl yeast RNA (Life Technologies), 7.5 % (v/v) glycerol, and 0.2 units SUPERase In RNase Inhibitor (Life Technologies) all in a total volume of 10 μl. Binding with or without unlabeled RsmW or RsmY as competitor RNA (5 pmol) was carried out for 30 min at 37 °C and then Bromophenol Blue was added (0.01 % wt/vol) before immediate electrophoresis on 6 % (w/v) non-denaturing polyacrylamide TBE gel (47 mM Tris, 45 mM boric acid, 1 mM EDTA, pH 8.3) at 4 °C. Imaging and image analyses were performed using a 9201 Odyssey Imaging System (LI-COR Biosciences) and Image Studio V5.0 software, respectively.

#### Statistical analysis

The paired Student’s *t*-test (two-tailed) was used. All statistical data were calculated using GraphPad Prism Software. Statistical significance was accepted when *P ≤ 0.05*.

## Abbreviations

3’-UTR, 3’-untranslated region; 5’RLM-RACE, RNA-Ligase Mediated-Rapid Amplification of cDNA Ends; 5’-UTR, 5’-untranslated region; BHI, Brain Heart Infusion; bp, base pair; H-NS, histone-like nucleoid structuring; HtpB, histidine-containing phosphorelay protein B; IHF, integration host factor; LB, Luria-Burtani lysogeny broth; log, logarithmic; nt, nucleotide; ON, overnight; ORF, open reading frame; PB, Peptone Broth; qRT-PCR, quantitative real-time PCR; RNA-seq, RNA sequencing; sRNAs, small non-coding regulatory RNAs; T3SS, type III secretion system; T6SS, type VI secretion system; TSB, tryptic soy broth; UAS, upstream activating sequence; Vfr, virulence factor regulator

## Additional files

Additional file 1: Figure S1. RsmW RNA levels increase in *P. aeruginosa* when grown at 37 °C versus 28 °C. Quantitative RT-PCR. (DOCX 67 kb) Additional file 2: Figure S2. (A) RsmA influences the RNA levels of PA4570 and RsmW. (B) RsmW levels decrease in the absence of the small RNA chaperone Hfq. Quantitative RT-PCR. (DOCX 48 kb) Additional file 3: Figure S3. PA4570 is unable to complement for RsmA or CsrA in biofilm production, swarming and glycogen synthesis. Biofilm, swarming, and glycogen synthesis assays of various *P. aeruginosa* and *E. coli* mutants. (DOCX 879 kb) Additional file 4: Figure S4. PA4570’s homology to RsmA and linkage to RsmW suggests mechanism for shifting the stoichiometric balance. Putative mechanistic model. (DOCX 130 kb) Additional file 5: Table S1. Genes differentially expressed in the Δ*rsmW* mutant compared to wild-type. Three cultures of each strain (*ΔrsmW* mutant and wild-type) were assessed by RNA-sequencing using the HiSeq 2000 platform (Illumina). Strains were grown to late stationary phase (16 hours) in Peptone Broth medium. (DOCX 35 kb) Additional file 6: Table S2. Strains and Plasmids used in this study. (DOCX 36 kb) Additional file 7: Table S3. Primers used in this study. (DOCX 18 kb)
