# Supplementary material for: RsmW, Pseudomonas aeruginosa small non-coding RsmA-binding RNA upregulated in biofilm versus planktonic growth conditions
Source: BMC Microbiol. 2016 Jul 19;16:155. doi: 10.1186/s12866-016-0771-y (PMC4950607; doi:10.1186/s12866-016-0771-y)
Supplement: Additional file 1: Figure S1. — RsmW RNA levels increase in P. aeruginosa when grown at 37 °C versus 28 °C. Quantitative RT-PCR. (DOCX 67 kb) [file 12866_2016_771_MOESM1_ESM.docx]

**Fig. S1. RsmW RNA levels increase in *P. aeruginosa* when grown at 37**°**C versus 28**°**C**. Quantitative RT-PCR of RNA harvested from *P. aeruginosa* grown for 16 hours, shaking in LB media at 28°C or 37°C. Assays were performed in triplicate and average fold changes are displayed. The values for all the samples were normalized relative to the value for 16S rRNA housekeeping gene. Error bars indicate s.e.m.
