# Supplementary material for: RsmW, Pseudomonas aeruginosa small non-coding RsmA-binding RNA upregulated in biofilm versus planktonic growth conditions
Source: BMC Microbiol. 2016 Jul 19;16:155. doi: 10.1186/s12866-016-0771-y (PMC4950607; doi:10.1186/s12866-016-0771-y)
Supplement: Additional file 2: Figure S2. — (A) RsmA influences the RNA levels of PA4570 and RsmW. (B) RsmW levels decrease in the absence of the small RNA chaperone Hfq. Quantitative RT-PCR. (DOCX 48 kb) [file 12866_2016_771_MOESM2_ESM.docx]

**Fig. S2. (A) RsmA influences the RNA levels of PA4570 and RsmW**. Quantitative RT-PCR fold changes of RsmW and PA4570 by comparing PASK10 grown in the absence of IPTG, (repressed *rsmA*) compared to PASK10 grown with 1 mM IPTG (induced *rsmA*) at mid-logarithmic growth phase. **(B) RsmW levels decrease in the absence of the small RNA chaperone Hfq**. Small regulatory RNAs PrrF1 and RsmY were used as a positive and negative control, respectively. Quantitative RT-PCR performed in triplicate displaying average fold changes. The values for all the samples were normalized relative to the value for *fabD* housekeeping gene. Error bars indicate s.e.m.
