# Supplementary material for: RsmW, Pseudomonas aeruginosa small non-coding RsmA-binding RNA upregulated in biofilm versus planktonic growth conditions
Source: BMC Microbiol. 2016 Jul 19;16:155. doi: 10.1186/s12866-016-0771-y (PMC4950607; doi:10.1186/s12866-016-0771-y)
Supplement: Additional file 3: Figure S3. — PA4570 is unable to complement for RsmA or CsrA in biofilm production, swarming and glycogen synthesis. Biofilm, swarming, and glycogen synthesis assays of various P. aeruginosa and E. coli mutants. (DOCX 879 kb) [file 12866_2016_771_MOESM3_ESM.docx]

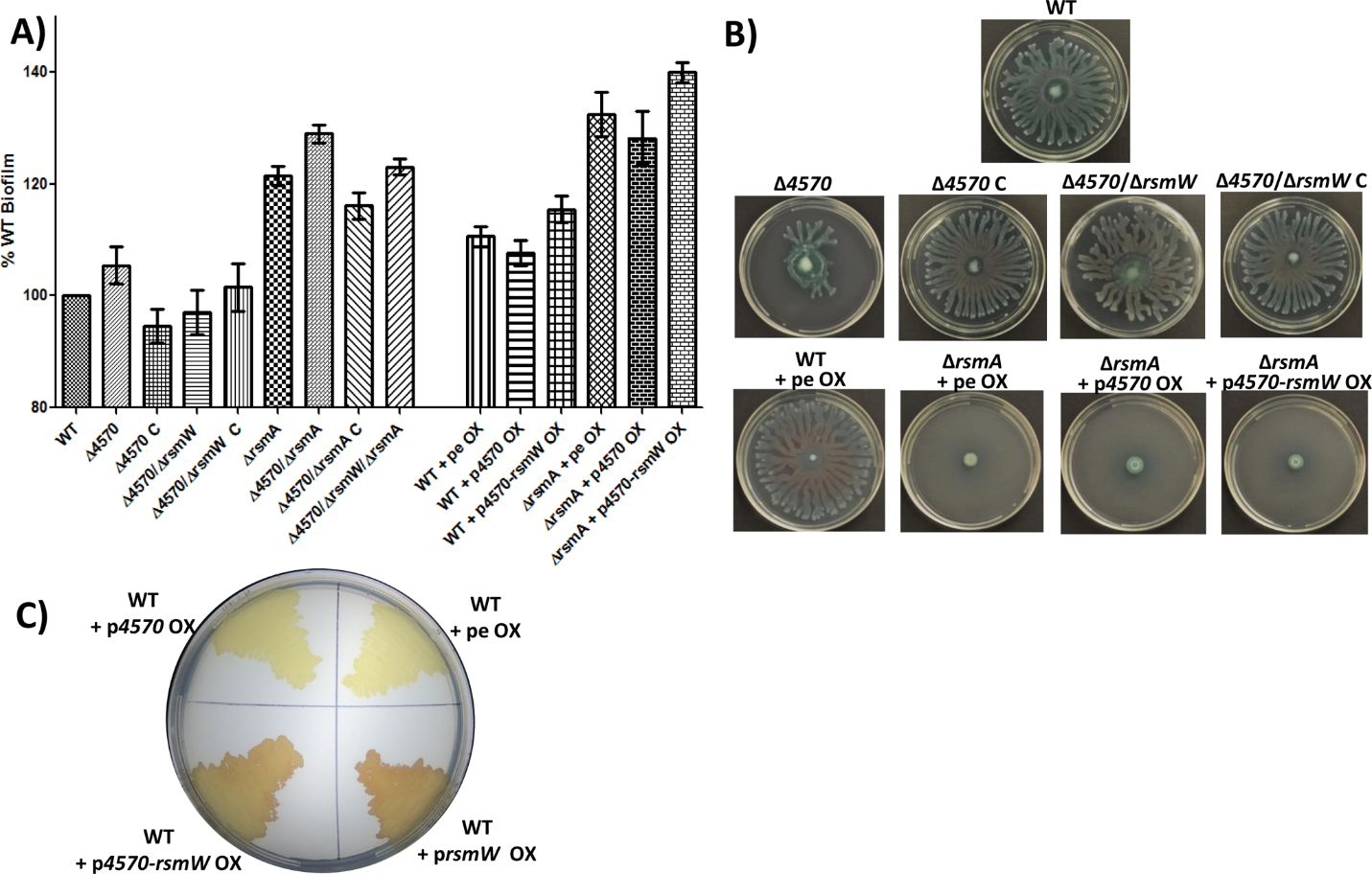


**Fig. S3. PA4570 is unable to complement for RsmA or CsrA in biofilm production, swarming and glycogen synthesis. (A)** **PA4570 or PA4570 with RsmW cannot complement a ∆*rsmA* mutant for biofilm development.** Assessment of biofilms was performed using 0.1% crystal violet staining method in 96-well microtiter plates after 24 hour of culture incubation at 37°C in LB. An OD550 nm reading was taken and the average of 5 experiments is shown with standard error depicted. Δ*4570*/Δ*rsmA* strain, but not Δ*4570*/Δ*rsmW*/Δ*rsmA* strain, showed an increase in biofilm production compared to the ∆*rsmA* mutant**.** ∆*rsmA* mutant overexpressing a plasmid containing PA4570 alone (p*4570* OX), but not PA4570 and RsmW (p*4570-rsmW* OX), demonstrated a modest decrease in biofilm production. **(B) Transforming the** **∆*rsmA* mutant with a plasmid overexpressing both PA4570 and RsmW or overexpressing PA4570 alone did not restore the swarming defect.** The ***∆****4570* mutant is also defective for swarming and swarming is restored when the PA4570+RsmW region is swapped back in *cis*. **(C) Glycogen synthesis is positively affected in *E. coli* overexpressing PA4570 with RsmW or RsmW alone but remains unaltered with overexpression of PA4570 alone.** Overproduction of glycogen on Kornberg medium is detectable by iodine staining giving a red appearance. Glycogen assay was assessed in wild-type *E. coli* carrying overexpression plasmids p*4570* OX, p*4570-rsmW* OX, p*rsmW* OX, and empty overexpression plasmid, pe OX. Abbreviations: C: WT “gene-swap”; OX: overexpression.
