# Supplementary material for: RsmW, Pseudomonas aeruginosa small non-coding RsmA-binding RNA upregulated in biofilm versus planktonic growth conditions
Source: BMC Microbiol. 2016 Jul 19;16:155. doi: 10.1186/s12866-016-0771-y (PMC4950607; doi:10.1186/s12866-016-0771-y)
Supplement: Additional file 4: Figure S4. — PA4570’s homology to RsmA and linkage to RsmW suggests mechanism for shifting the stoichiometric balance. Putative mechanistic model. (DOCX 130 kb) [file 12866_2016_771_MOESM4_ESM.docx]

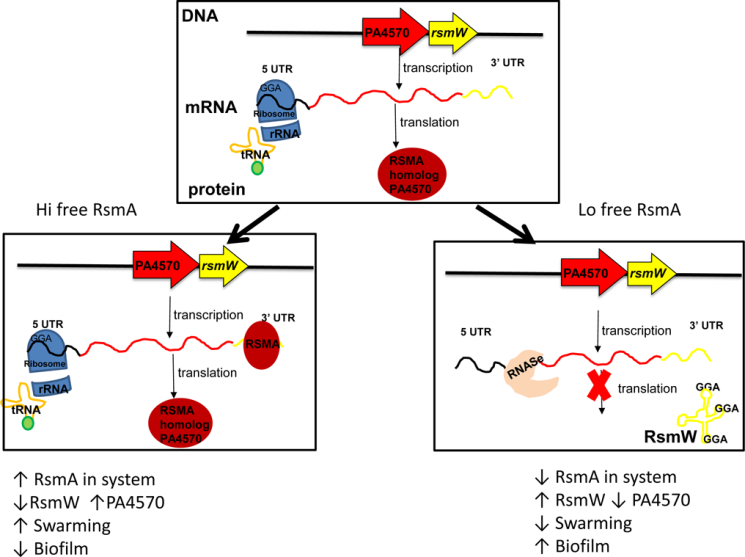


**Fig. S4. PA4570’s homology to RsmA and linkage to RsmW suggests mechanism for shifting the stoichiometric balance**. High free levels of RsmA bind RsmW and stabilize PA4570, leading to an increase in PA4570 expression and an increase in RsmA/RsmN/PA4570 regulation. In the absence of free RsmA, the PA4570-*rsmW* transcript is not bound by RsmA leading to an increase in RsmW to further deplete the free RsmA/RsmN/PA4570 population**.**
