# Supplementary material for: RsmW, Pseudomonas aeruginosa small non-coding RsmA-binding RNA upregulated in biofilm versus planktonic growth conditions
Source: BMC Microbiol. 2016 Jul 19;16:155. doi: 10.1186/s12866-016-0771-y (PMC4950607; doi:10.1186/s12866-016-0771-y)
Supplement: Additional file 5: Table S1. — Genes differentially expressed in the ΔrsmW mutant compared to wild-type. Three cultures of each strain (ΔrsmW mutant and wild-type) were assessed by RNA-sequencing using the HiSeq 2000 platform (Illumina). Strains were grown to late stationary phase (16 hours) in Peptone Broth medium. (DOCX 35 kb) [file 12866_2016_771_MOESM5_ESM.docx]

Table S1. Genes differentially expressed in the Δ*rsmW* mutant compared to wild-type.

| **ORF** | **FC** | **Padj.** | **ORF description** |
| --- | --- | --- | --- |
| **Defense and Virulence** | | | |
| PA0459 | 1.39 | 3.7E-02 | probable ClpA/B protease ATP binding subunit |
| PA0779 | -1.6 | 1.8E-03 | probable ATP-dependent protease |
| PA1871 | -1.8 | 1.1E-06 | LasA protease precursor ***** |
| PA1877 | 1.54 | 3.2E-02 | probable secretion protein |
| PA2194 | -2.4 | 1.7E-08 | hydrogen cyanide synthase HcnB |
| PA2195 | -1.8 | 4.5E-08 | hydrogen cyanide synthase HcnC |
| PA2570 | -1.7 | 3.3E-07 | LecA |
| PA3724 | -2.1 | 5.2E-11 | elastase LasB ***** |
| PA0807 | -4 | 3.7E-02 | AmpDh3 |
| PA0867 | -1.7 | 1.6E-03 | membrane-bound lysozyme inhibitor of c-type lysozyme MliC |
| PA0984 | -1.7 | 2.1E-02 | colicin immunity protein |
| PA1596 | -2.1 | 7.2E-05 | heat shock protein HtpG |
| PA3126 | -2.1 | 1.7E-02 | heat-shock protein IbpA |
| PA3161 | -2.9 | 8.0E-03 | integration host factor beta subunit, himD |
| PA3450 | 2.22 | 4.9E-02 | probable antioxidant protein |
| PA3523 | 7.75 | 4.6E-02 | RND efflux membrane fusion protein precursor * |
| PA3721 | 1.53 | 3.1E-03 | NalC |
| PA4208 | 3.19 | 5.3E-05 | probable outer membrane protein precursor, ompD |
| **Regulators** | | | |
| PA0436 | 1.63 | 2.6E-04 | probable transcriptional regulator |
| PA0535 | 2.48 | 1.6E-02 | probable transcriptional regulator |
| PA0652 | 1.62 | 5.1E-03 | transcriptional regulator Vfr |
| PA1504 | -83 | 1.7E-02 | probable transcriptional regulator |
| PA1630 | -1.9 | 1.5E-02 | probable transcriptional regulator |
| PA1961 | 4.01 | 2.5E-02 | probable transcriptional regulator |
| PA2100 | -2 | 4.5E-06 | probable transcriptional regulator |
| PA2126 | 1.73 | 2.2E-02 | conserved hypothetical protein, CgrC |
| PA2127 | 1.94 | 1.1E-05 | conserved hypothetical protein, CgrA |
| PA2299 | 1.83 | 5.7E-03 | probable transcriptional regulator |
| PA2312 | 2.38 | 5.2E-03 | probable transcriptional regulator |
| PA2359 | 2.43 | 1.4E-02 | probable transcriptional regulator |
| PA2885 | -1.8 | 8.2E-03 | putative repressor of atu genes |
| PA3124 | -2 | 2.0E-06 | probable transcriptional regulator |
| PA3363 | 1.72 | 2.1E-02 | aliphatic amidase regulator, AmiR |
| PA3477 | -1.7 | 3.2E-07 | transcriptional regulator RhlR ***** |
| PA3879 | 1.78 | 8.8E-03 | two-component response regulator NarL |
| PA3965 | -2.5 | 1.2E-02 | probable transcriptional regulator |
| PA4070 | -1.8 | 5.7E-03 | probable transcriptional regulator |
| PA4147 | 1.59 | 4.2E-03 | transcriptional regulator AcoR |
| PA4726 | 1.53 | 6.3E-04 | two-component response regulator CbrB |
| PA5301 | -1.7 | 1.7E-07 | probable transcriptional regulator |
| PA5356 | -2.1 | 1.1E-05 | transcriptional regulator, GlcC |
| **c-di-GMP** | | | |
| PA5483 | -2.7 | 3.6E-03 | two-component response regulator AlgB |
| PA2567 | 1.56 | 1.3E-02 | hypothetical protein, c-di-GMP phoshodiesterase |
| PA3885 | 84.6 | 1.8E-02 | protein tyrosine phosphatase TpbA |
| PA4601 | 2.08 | 2.8E-09 | motility regulator, MorA |
| **Iron homeostasis** | | | |
| PA0149 | 2.44 | 1.8E-02 | probable sigma-70 factor, ECF subfamily |
| PA0150 | 2.16 | 2.3E-02 | probable transmembrane sensor |
| PA0929 | 1.38 | 1.8E-02 | two-component response regulator |
| PA1300 | 1.62 | 3.1E-05 | probable sigma-70 factor, ECF subfamily |
| PA1301 | 1.54 | 2.5E-04 | probable transmembrane sensor |
| PA2093 | 2.4 | 2.7E-03 | probable sigma-70 factor, ECF subfamily |
| PA2094 | 3.02 | 7.3E-11 | probable transmembrane sensor |
| PA2398 | -1.6 | 1.9E-02 | ferripyoverdine receptor |
| PA2686 | 1.94 | 1.7E-04 | two-component response regulator PfeR |
| PA2687 | 1.86 | 3.5E-06 | two-component sensor PfeS |
| PA3285 | 1.54 | 5.1E-03 | probable sigma-70 factor, ECF subfamily |
| PA4159 | 1.83 | 9.5E-06 | ferrienterobactin-binding periplasmic protein precursor FepB |
| PA4160 | 2.59 | 2.2E-05 | ferric enterobactin transport protein FepD |
| PA4161 | 4.1 | 5.2E-03 | ferric enterobactin transport protein FepG |
| PA4210 | -1.8 | 4.6E-03 | probable phenazine biosynthesis protein, phzA1 |
| PA4223 | 1.6 | 5.9E-04 | probable ATP-binding component of ABC transporter |
| PA1905 | 1.54 | 4.9E-02 | probable pyridoxamine 5'-phosphate oxidase, phzG2 |
| **Motility** | | | |
| PA1092 | -1.6 | 1.7E-02 | flagellin type B |
| PA1094 | -1.5 | 1.7E-03 | flagellar capping protein FliD |
| PA2654 | -1.9 | 1.3E-02 | probable chemotaxis transducer |
| PA2788 | -1.5 | 7.0E-04 | probable chemotaxis transducer |
| PA3479 | -1.5 | 4.0E-04 | rhamnosyltransferase chain A, RhlA |
| PA4290 | -1.7 | 1.3E-03 | probable chemotaxis transducer |
| PA4306 | 2.68 | 4.2E-08 | Type IVb pilin, Flp |
| PA4649 | 86 | 1.7E-02 | cupE2 |
| PA5276 | 1.73 | 4.8E-02 | Lipopeptide LppL precursor |
| **LPS, Cell Envelope** | | | |
| PA1385 | -1.6 | 4.3E-03 | probable glycosyl transferase |
| PA3337 | 1.95 | 1.7E-03 | ADP-L-glycero-D-mannoheptose 6-epimerase |
| PA3636 | -1.8 | 2.3E-03 | 2-dehydro-3-deoxyphosphooctonate aldolase |
| PA4414 | 1.51 | 7.7E-03 | UDP-N-acetylmuramoylalanine--D-glutamate ligase |
| **Transporters** | | | |
| PA0287 | 2.62 | 4.9E-03 | 3-guanidinopropionate transport protein |
| PA0811 | 2.73 | 3.4E-02 | probable major facilitator superfamily (MFS) transporter |
| PA1493 | 1.7 | 2.7E-02 | sulfate-binding protein of ABC transporter |
| PA1549 | 1.86 | 4.1E-04 | probable cation-transporting P-type ATPase |
| PA1964 | 1.76 | 1.2E-03 | probable ATP-binding component of ABC transporter |
| PA2092 | 2.32 | 3.4E-08 | probable major facilitator superfamily (MFS) transporter |
| PA2327 | 1.86 | 4.4E-06 | probable permease of ABC transporter |
| PA2997 | -1.8 | 3.7E-02 | Na+-translocating NADH:ubiquinone oxidoreductase subunit Nrq3 |
| PA3234 | -1.9 | 6.2E-06 | probable sodium:solute symporter |
| PA3315 | -2.6 | 4.9E-02 | probable permease of ABC transporter |
| PA3447 | 3.29 | 4.1E-04 | probable ATP-binding component of ABC transporter |
| PA3766 | -1.9 | 6.2E-03 | probable aromatic amino acid transporter |
|  |  |  |  |
| **ORF** | **FC** | **P_adj._** | **ORF description** |
| **Transporters Cont.** | | | |
| PA3839 | 2.06 | 2.5E-09 | probable sodium:sulfate symporter |
| PA3920 | 2.68 | 2.1E-06 | probable metal transporting P-type ATPase |
| PA4156 | 2.14 | 1.1E-12 | probable TonB-dependent receptor |
| PA4223 | 1.6 | 5.9E-04 | probable ATP-binding component of ABC transporter |
| PA4276 | 1.93 | 1.2E-02 | secretion protein SecE |
| PA4358 | 2.1 | 2.3E-11 | probable ferrous iron transport protein |
| PA4503 | -1.7 | 1.8E-02 | probable permease of ABC transporter |
| PA4747 | 1.51 | 1.8E-02 | secretion protein SecG |
| PA5097 | 2.01 | 1.6E-02 | probable amino acid permease |
| PA5207 | 2.23 | 1.6E-03 | probable phosphate transporter |
| PA5230 | 2.6 | 3.6E-18 | probable permease of ABC transporter |
| PA5231 | 2.11 | 5.2E-11 | probable ATP-binding/permease fusion ABC transporter |
| PA5501 | 3.03 | 9.5E-09 | permease of ABC zinc transporter ZnuB |
| **Carbon Utilization: TCA, Glycolysis, Gluconeogensis, Acetate Metabolism** | | | |
| PA0106 | 0.55 | 5.0E-04 | cytochrome c oxidase, subunit I |
| PA0211 | 2.39 | 8.7E-05 | malonate decarboxylase beta subunit |
| PA0212 | 2.22 | 9.3E-04 | malonate decarboxylase gamma subunit |
| PA0214 | 2.37 | 2.7E-03 | probable acyl transferase |
| PA0215 | 1.74 | 2.8E-02 | malonate transporter MadL |
| PA0299 | -1.7 | 1.9E-06 | putrescine aminotransferase |
| PA0304 | -1.5 | 7.9E-03 | polyamine transport protein PotI |
| PA0795 | -1.6 | 3.2E-02 | citrate synthase 2 |
| PA0835 | 1.69 | 4.5E-04 | phosphate acetyltransferase |
| PA0836 | 1.65 | 2.8E-03 | acetate kinase |
| PA0840 | 4.74 | 1.5E-02 | probable oxidoreductase |
| PA0852 | -1.6 | 1.1E-03 | chitin-binding protein CbpD precursor |
| PA1546 | 1.52 | 5.9E-04 | oxygen-independent coproporphyrinogen III oxidase |
| PA1581 | 3.07 | 1.2E-04 | succinate dehydrogenase (C subunit) |
| PA1856 | -1.6 | 4.4E-02 | probable cytochrome oxidase subunit |
| PA1947 | -1.7 | 4.8E-04 | ribose transport protein RbsA |
| PA2119 | 2.16 | 9.2E-10 | alcohol dehydrogenase (Zn-dependent) |
| PA2298 | 2.4 | 8.6E-08 | probable oxidoreductase |
| PA2300 | -2 | 4.7E-11 | Chitinase chiC |
| PA2304 | -1.8 | 1.4E-04 | AmbC |
| PA2552 | 2.85 | 8.6E-03 | probable acyl-CoA dehydrogenase |
| PA2624 | 1.58 | 9.3E-04 | isocitrate dehydrogenase |
| PA3132 | -3.4 | 8.3E-04 | probable hydrolase |
| PA3366 | 3.18 | 5.2E-28 | aliphatic amidase |
| PA3972 | 1.88 | 1.0E-07 | probable acyl-CoA dehydrogenase |
| PA4329 | 1.9 | 4.1E-04 | pyruvate kinase II |
| PA4429 | -1.8 | 1.2E-02 | probable cytochrome c1 precursor |
| PA4571 | 2.53 | 5.6E-06 | probable cytochrome c |
| PA4587 | 3.18 | 1.5E-11 | cytochrome c551 peroxidase precursor |
| PA4889 | 2.88 | 7.5E-03 | probable oxidoreductase |
| PA5312 | -1.6 | 7.4E-04 | probable aldehyde dehydrogenase |
| PA5427 | 2.14 | 4.8E-12 | alcohol dehydrogenase |
| PA5432 | -2 | 5.5E-03 | probable acetyltransferase |
| PA5554 | -1.9 | 3.9E-07 | ATP synthase beta chain |
| PA5555 | -2.1 | 2.3E-04 | ATP synthase gamma chain |
| PA5556 | -1.9 | 3.2E-08 | ATP synthase alpha chain |
| PA5557 | -2.2 | 6.1E-09 | ATP synthase delta chain |
| PA5558 | -2.2 | 3.2E-10 | ATP synthase B chain |
| PA5559 | -2.3 | 7.6E-05 | atp synthase C chain |
| PA5560 | -2 | 4.7E-03 | ATP synthase A chain |
| **Fatty Acid Metabolism** | | | |
| PA0447 | 1.91 | 3.2E-09 | glutaryl-CoA dehydrogenase |
| PA1609 | 1.73 | 6.3E-04 | beta-ketoacyl-ACP synthase I |
| PA1629 | -2.9 | 4.1E-02 | probable enoyl-CoA hydratase/isomerase |
| PA1828 | -3 | 4.1E-02 | probable short-chain dehydrogenase |
| PA4888 | 3.77 | 1.5E-12 | acyl-CoA delta-9-desaturase, DesB |
| PA5031 | -108 | 2.1E-03 | probable short chain dehydrogenase |
| PA5521 | -1.9 | 1.1E-08 | probable short-chain dehydrogenase |
| **Amino Acid Metabolism** | | | |
| PA0196 | 0.41 | 1.5E-02 | pyridine nucleotide transhydrogenase, beta subunit |
| PA0265 | 0.53 | 2.0E-06 | succinate-semialdehyde dehydrogenase |
| PA0530 | 3.81 | 5.9E-07 | probable class III pyridoxal phosphate-dependent aminotransferase |
| PA0531 | 3.51 | 8.1E-06 | probable glutamine amidotransferase ***** |
| PA0746 | -2.2 | 9.0E-03 | probable acyl-CoA dehydrogenase |
| PA0747 | -1.6 | 1.1E-03 | probable aldehyde dehydrogenase |
| PA0865 | -1.9 | 1.2E-07 | 4-hydroxyphenylpyruvate dioxygenase |
| PA1156 | -1.5 | 4.0E-02 | NrdA, catalytic component of class Ia ribonucleotide reductase |
| PA1535 | -2 | 2.8E-03 | probable acyl-CoA dehydrogenase |
| PA2008 | -1.8 | 4.9E-02 | fumarylacetoacetase |
| PA2080 | 5.21 | 7.9E-06 | kynureninase KynU ***** |
| PA2084 | 2.03 | 6.2E-04 | probable asparagine synthetase |
| PA2247 | -1.8 | 3.3E-05 | 2-oxoisovalerate dehydrogenase (alpha subunit) |
| PA2248 | -2 | 9.4E-07 | 2-oxoisovalerate dehydrogenase (beta subunit) |
| PA2249 | -1.9 | 3.4E-07 | branched-chain alpha-keto acid dehydrogenase (lipoamide component) |
| PA2250 | -1.6 | 3.6E-02 | lipoamide dehydrogenase-Val |
| PA2445 | 1.69 | 1.6E-02 | glycine cleavage system protein P2 |
| PA3120 | -3.1 | 8.7E-04 | 3-isopropylmalate dehydratase small subunit |
| PA3121 | -2.3 | 3.3E-11 | 3-isopropylmalate dehydratase large subunit |
| PA3568 | -2.4 | 1.1E-13 | probable acetyl-coa synthetase |
| PA3569 | -1.9 | 4.5E-08 | 3-hydroxyisobutyrate dehydrogenase |
| PA3570 | -1.5 | 4.8E-04 | methylmalonate-semialdehyde dehydrogenase |
| PA3792 | -9.7 | 4.8E-12 | 2-isopropylmalate synthase, leuA |
| PA4694 | -2 | 1.5E-04 | ketol-acid reductoisomerase |
| PA4695 | -2.6 | 5.8E-09 | acetolactate synthase isozyme III small subunit |
| PA4696 | -1.7 | 1.9E-03 | acetolactate synthase large subunit |
| PA4749 | 1.5 | 3.5E-03 | phosphoglucosamine mutase |
| PA5173 | 2.16 | 1.5E-04 | carbamate kinase |
| PA5277 | 1.76 | 2.0E-03 | diaminopimelate decarboxylase |
| PA5522 | -1.7 | 4.7E-04 | probable glutamine synthetase |
| PA5541 | 2.81 | 4.7E-02 | dihydroorotase |

Table S1 continued. Genes differentially expressed in the Δ*rsmW* mutant compared to wild-type.

| **ORF** | **FC** | **Padj** | **ORF description** |
| --- | --- | --- | --- |
| **Hypothetical** | | | |
| PA0039 | -1.52 | 1.3E-02 | hypothetical protein |
| PA0123 | -2.45 | 4.5E-02 | probable transcriptional regulator |
| PA0124 | -6.22 | 6.1E-06 | hypothetical protein |
| PA0125 | -8.58 | 1.5E-02 | hypothetical protein |
| PA0141 | 1.89 | 3.0E-06 | conserved hypothetical protein |
| PA0201 | 3.65 | 3.3E-11 | hypothetical protein |
| PA0481 | -2.01 | 8.4E-03 | hypothetical protein |
| PA0488 | -4.02 | 3.2E-02 | conserved hypothetical protein |
| PA0529 | 2.54 | 2.9E-12 | conserved hypothetical protein |
| PA0613 | -2.78 | 4.7E-03 | hypothetical protein |
| PA0624 | -2.06 | 2.4E-04 | hypothetical protein |
| PA0625 | -1.99 | 2.5E-09 | hypothetical protein |
| PA0626 | -1.80 | 1.1E-02 | hypothetical protein |
| PA0628 | -2.66 | 7.3E-11 | conserved hypothetical protein |
| PA0633 | -2.82 | 4.8E-12 | hypothetical protein |
| PA0634 | -2.42 | 3.0E-07 | hypothetical protein |
| PA0635 | -2.46 | 1.0E-03 | hypothetical protein |
| PA0636 | -2.18 | 2.9E-09 | hypothetical protein |
| PA0637 | -2.03 | 1.8E-02 | conserved hypothetical protein |
| PA0643 | -1.69 | 7.5E-03 | hypothetical protein |
| PA0882 | 3.60 | 1.3E-02 | hypothetical protein |
| PA0935 | -1.64 | 3.2E-05 | conserved hypothetical protein |
| PA0935 | -1.64 | 3.2E-05 | conserved hypothetical protein |
| PA0952 | -2.66 | 1.1E-03 | hypothetical protein |
| PA0957 | 1.65 | 4.4E-02 | hypothetical protein |
| PA1041 | -2.03 | 6.9E-11 | probable outer membrane protein precursor |
| PA1060 | 1.57 | 2.7E-02 | hypothetical protein |
| PA1063 | 2.31 | 5.1E-05 | hypothetical protein |
| PA1190 | -1.76 | 3.0E-04 | conserved hypothetical protein |
| PA1197 | 1.54 | 1.2E-02 | hypothetical protein |
| PA1232 | -4.19 | 4.1E-02 | hypothetical protein |
| PA1442 | -1.60 | 1.1E-02 | conserved hypothetical protein |
| PA1517 | -2.26 | 8.8E-03 | conserved hypothetical protein |
| PA1527 | 1.56 | 2.6E-04 | conserved hypothetical protein |
| PA1545 | 0.66 | 9.2E-03 | hypothetical protein |
| PA1550 | 1.73 | 2.8E-06 | hypothetical protein |
| PA1572 | 1.34 | 2.8E-02 | conserved hypothetical protein |
| PA1789 | 1.60 | 3.7E-04 | hypothetical protein |
| PA1814 | 1.53 | 5.7E-03 | hypothetical protein |
| PA1830 | -1.87 | 1.9E-03 | hypothetical protein |
| PA1866 | 1.54 | 3.2E-03 | hypothetical protein |
| PA1875 | 1.51 | 4.7E-02 | probable outer membrane protein precursor |
| PA2026 | -3.46 | 1.3E-04 | conserved hypothetical protein |
| PA2043 | 2.22 | 4.0E-03 | hypothetical protein |
| PA2070 | 1.81 | 3.1E-02 | hypothetical protein |
| PA2072 | -1.58 | 1.6E-02 | conserved hypothetical protein |
| PA2090 | 2.28 | 2.1E-04 | hypothetical protein |
| PA2091 | 2.17 | 4.9E-06 | hypothetical protein |
| PA2146 | 1.85 | 4.3E-03 | conserved hypothetical protein |
| PA2274 | 2.00 | 6.5E-05 | hypothetical protein |
| PA2309 | 1.92 | 2.8E-02 | hypothetical protein |
| PA2310 | 2.30 | 2.1E-02 | hypothetical protein |
| PA2328 | 1.91 | 1.6E-04 | hypothetical protein |
| PA2330 | 4.98 | 2.1E-10 | hypothetical protein |
| PA2412 | -1.56 | 3.2E-03 | conserved hypothetical protein |
| PA2564 | -1.63 | 1.9E-05 | hypothetical protein |
| PA2753 | 1.36 | 3.8E-02 | hypothetical protein |
| PA2805 | 1.51 | 1.1E-03 | hypothetical protein |
| PA2854 | 1.55 | 3.6E-04 | conserved hypothetical protein |
| PA3070 | 1.77 | 1.5E-02 | conserved hypothetical protein |
| PA3091 | -1.67 | 1.6E-02 | hypothetical protein |
| PA3119 | -7.70 | 2.8E-03 | conserved hypothetical protein |
| PA3202 | 1.79 | 8.3E-04 | conserved hypothetical protein |
| PA3229 | -2.24 | 9.9E-08 | hypothetical protein |
| PA3235 | -3.07 | 5.2E-11 | conserved hypothetical protein |
| PA3267 | 1.51 | 2.5E-02 | hypothetical protein |
| PA3465 | 1.69 | 1.7E-04 | conserved hypothetical protein |
| PA3496 | -1.99 | 4.3E-04 | hypothetical protein |
| PA3519 | 2.18 | 1.8E-03 | hypothetical protein |
| PA3520 | 2.21 | 8.4E-09 | hypothetical protein |
| PA3578 | 1.55 | 6.1E-03 | conserved hypothetical protein |
| PA3600 | 2.12 | 3.3E-09 | conserved hypothetical protein |
| PA3601 | 2.11 | 1.3E-10 | conserved hypothetical protein |
| PA3613 | 1.50 | 3.8E-04 | hypothetical protein |
| PA3614 | 1.77 | 3.7E-06 | hypothetical protein |
|  |  |  |  |
| **ORF** | **FC** | **Padj** | **ORF description** |
| **Hypothetical Cont.** | | | |
| PA3691 | 1.61 | 5.0E-02 | hypothetical protein |
| PA3851 | 1.60 | 3.5E-02 | hypothetical protein |
| PA3939 | 3.80 | 3.4E-08 | hypothetical protein |
| PA3983 | 1.65 | 2.7E-02 | conserved hypothetical protein |
| PA4045 | 3.57 | 2.2E-08 | conserved hypothetical protein |
| PA4046 | 2.36 | 1.1E-03 | hypothetical protein |
| PA4062 | 2.46 | 3.7E-04 | hypothetical protein |
| PA4063 | 2.97 | 7.8E-05 | hypothetical protein |
| PA4065 | 2.22 | 4.8E-03 | hypothetical protein |
| PA4154 | 8.85 | 2.1E-02 | conserved hypothetical protein |
| PA4155 | 3.18 | 5.0E-08 | hypothetical protein |
| PA4328 | 1.57 | 2.9E-02 | hypothetical protein |
| PA4348 | 1.51 | 2.8E-03 | conserved hypothetical protein |
| PA4352 | 1.78 | 2.1E-03 | conserved hypothetical protein |
| PA4357 | 1.96 | 6.2E-09 | conserved hypothetical protein |
| PA4359 | 2.07 | 1.6E-02 | conserved hypothetical protein |
| PA4377 | -1.52 | 1.9E-02 | hypothetical protein |
| PA4377 | -1.52 | 1.9E-02 | hypothetical protein |
| PA4465 | 1.59 | 3.6E-03 | conserved hypothetical protein |
| PA4570 | -2.29 | 5.3E-05 | hypothetical protein |
| PA4577 | 2.29 | 3.5E-02 | hypothetical protein |
| PA4607 | -1.88 | 6.6E-09 | hypothetical protein |
| PA4607 | -1.88 | 6.6E-09 | hypothetical protein |
| PA4667 | 1.58 | 1.4E-02 | hypothetical protein |
| PA4674 | -1.80 | 1.2E-05 | conserved hypothetical protein |
| PA4718 | 1.86 | 3.1E-02 | hypothetical protein |
| PA4718 | 1.86 | 3.1E-02 | hypothetical protein |
| PA4757 | 1.57 | 3.8E-02 | conserved hypothetical protein |
| PA4803 | -1.88 | 3.3E-02 | hypothetical protein |
| PA4916 | 1.56 | 1.6E-03 | hypothetical protein |
| PA4961 | 1.65 | 3.8E-03 | hypothetical protein |
| PA5007 | 2.24 | 1.6E-02 | hypothetical protein |
| PA5025 | 2.27 | 1.1E-02 | homocysteine synthase |
| PA5026 | 2.12 | 3.2E-02 | hypothetical protein |
| PA5027 | 2.24 | 4.5E-04 | hypothetical protein |
| PA5137 | -1.85 | 9.7E-04 | hypothetical protein |
| PA5182 | -2.08 | 2.8E-02 | hypothetical protein |
| PA5247 | 1.50 | 2.8E-02 | conserved hypothetical protein |
| PA5314 | -2.06 | 5.2E-05 | hypothetical protein |
| PA5392 | -89.88 | 9.6E-03 | conserved hypothetical protein |
| PA5460 | 2.69 | 1.4E-04 | hypothetical protein |
| PA5475 | 1.55 | 3.0E-04 | hypothetical protein |
| PA5487 | 1.52 | 4.8E-04 | hypothetical protein |
| **Bacteriophage** | | | |
| PA0618 | -2.4 | 1.9E-08 | probable bacteriophage protein |
| PA0619 | -1.9 | 1.3E-02 | probable bacteriophage protein |
| PA0620 | -2.1 | 3.2E-07 | probable bacteriophage protein |
| PA0621 | -2 | 4.5E-02 | conserved hypothetical protein |
| PA0622 | -2.6 | 1.1E-04 | probable bacteriophage protein |
| PA0623 | -2.6 | 9.2E-10 | probable bacteriophage protein |
| **Replication, Translation** | | | |
| PA0963 | -1.9 | 4.3E-05 | aspartyl-tRNA synthetase |
| PA1886 | 1.55 | 2.1E-02 | DNA polymerase II |
| PA2744 | 1.76 | 8.8E-07 | threonyl-tRNA synthetase |
| PA3742 | -1.5 | 4.1E-02 | 50S ribosomal protein L19 |
| PA3802 | 0.59 | 7.1E-03 | histidyl-tRNA synthetase |
| PA4078 | -1.9 | 5.8E-09 | probable nonribosomal peptide synthetase |
| PA4254 | -1.8 | 6.1E-03 | 30S ribosomal protein S17 |
| PA4256 | -1.6 | 3.8E-03 | 50S ribosomal protein L16 |
| PA4271 | -1.8 | 5.6E-08 | 50S ribosomal protein L7 / L12 |
| PA5051 | 1.73 | 3.0E-03 | arginyl-tRNA synthetase |
| **Misc.** | | | |
| PA0837 | 1.58 | 1.4E-04 | peptidyl-prolyl cis-trans isomerase SlyD |
| PA2062 | 1.9 | 3.7E-06 | probable pyridoxal-phosphate dependent enzyme |
| PA3365 | 2.9 | 2.5E-07 | probable chaperone |
| PA3529 | -1.6 | 1.5E-03 | probable peroxidase |
| PA3737 | 1.54 | 1.4E-02 | thiol:disulfide interchange protein DsbC |
| PA4047 | 1.51 | 1.8E-02 | GTP cyclohydrolase II |
| PA4466 | 1.73 | 2.9E-03 | probable phosphoryl carrier protein |
| PA5066 | 1.55 | 4.2E-03 | phosphoribosyl-AMP cyclohydrolase |
| PA5098 | 3.53 | 1.6E-03 | histidine ammonia-lyase |
| PA5147 | -1.8 | 3.2E-02 | A / G specific adenine glycosylase |
| PA5243 | 1.6 | 2.2E-04 | delta-aminolevulinic acid dehydratase |
| PA5259 | 1.74 | 4.5E-05 | uroporphyrinogen-III synthetase |
| PA5564 | -1.8 | 4.9E-02 | glucose inhibited division protein B |

a. Strains were grown to late stationary phase (16 hours) in Peptone Broth medium.

b. Three cultures of each strain (*ΔrsmW* mutant and wild-type) were assessed by RNA-sequencing using the HiSeq 2000 platform (Illumina).

c. Differential expression analyses were assessed using the Bioconductor software package, DEseq.

d. Genes $\geq$ 1.5 fold change (FC) in expression and P_adj_ $\leq$0.05 are reported.

e. (*) RNA-seq expression trends confirmed by qRT-PCR on select ORFs using 16S as housekeeping ORF and reported as fold change (FC) +

standard error: [*rhlR* (FC= -2.73 + 0.2); *lasA* (FC= -1.73 + 0.3); *lasB* (FC= -1.82 + 0.4); PA0531 (FC= 1.59 + 0.1); PA2080 (FC= 5.51 + 0.4); and

PA3523 (FC= 1.66 + 0.2)].

f. Table abbreviations: ORF- Open reading frame; FC- fold change; P_adj_- adjusted *P* value.
