# Supplementary material for: RsmW, Pseudomonas aeruginosa small non-coding RsmA-binding RNA upregulated in biofilm versus planktonic growth conditions
Source: BMC Microbiol. 2016 Jul 19;16:155. doi: 10.1186/s12866-016-0771-y (PMC4950607; doi:10.1186/s12866-016-0771-y)
Supplement: Additional file 6: Table S2. — Strains and Plasmids used in this study. (DOCX 36 kb) [file 12866_2016_771_MOESM6_ESM.docx]

Table S2. Strains and Plasmids used in this study.

|  | **Description** | **Source** |
| --- | --- | --- |
| **Strains** | | |
| PAO1-UW | Wild-type, University of Washington subline | [[1](#_ENREF_1)] |
| PW5341 | *gacA*-E08::ISphoA/hah | [[1](#_ENREF_1)] |
| PW6883 | *rhlR*-H04::ISphoA/hah | [[1](#_ENREF_1)] |
| PAO1-N | Wild-type, University of Nottingham subline from which PAZH13 and PASK10 are derived | [[2](#_ENREF_2)] |
| Δ*rsmW* | PAO1-N / *rsmW* deletion mutant, Gm^r^ | This study |
| PASC659 | (Δ*rsmYZ*) rsmY/rsmZ double deletion mutant | unpublished, Heeb et al. |
| Δ*rsmWYZ* | PASC659 / *rsmW* deletion mutant; *rsmW*/rsmY/rsmZ triple deletion mutant, Gm^r^ | This study |
| Δ*rsmWYZ* C | *rsmWYZ* triple mutant strain with *rsmW* WT gene swapped back in *cis,* *rsmW* ^-/+^ , St^r^ | This study |
| WT + pe OX | PAO1-N + pJAK12; St^r^ | This study |
| WT + p*rsmW* OX | PAO1-N + pJAK12-RBS + *rsmW* (5118199-5118345) ; St^r^ | This study |
| Δ*rsmYZ* + pe OX | PASC659 + pJAK12; St^r^ | This study |
| Δ*rsmYZ* + p*rsmW* OX | PASC659 + pJAK12-RBS + *rsmW* (5118199-5118345) ; St^r^ | This study |
| Δ*4570* | PAO1-N/ *PA4570* deletion mutant, Gm^r^ | This study |
| Δ*4570* C | PAO1-N/ *PA4570* deletion mutant *cis*-complement strain, *PA4570*^—/+^ ,St^r^ | This study |
| Δ*4570* / Δ*rsmW* | Δ*4570*/ *rsmW* deletion mutant, Gm^r^ | This study |
| Δ*4570* / Δ*rsmW* C | Δ*4570*/ *rsmW* deletion mutant strain with *PA4570-rsmW* WT region swapped back in *cis*, *PA44570-rsmW^-^*^/+^ ,St^r^ | This study |
| PAZH13 | (Δ*rsmA*); *rsmA* in-frame deletion mutant | [[3](#_ENREF_3)] |
| Δ*rsmA*/ Δ*4570* | PAZH13/ *PA4570* deletion mutant,Gm^r^ | This study |
| Δ*rsmA*/ Δ*4570* C | PAZH13/ *PA4570* deletion double mutant *-* strain with *PA4570* WT region swapped back in *cis*, *PA4570*^—/+^ ,St^r^ | This study |
| Δ*rsmA*/ Δ*4570* / Δ*rsmW* | PAZH13/ *PA4570* and *rsmW* deletion triple mutant, Gm^r^ | This study |
| WT + p*4570* OX | PAO1-N + pJAK12 + *PA4570* (5117971-5118212) ; St^r^ | This study |
| WT + p*4570*- *rsmW* OX | PAO1-N + pJAK12 + *PA4570*-*rsmW* ( 5117971-5118537); St^r^ | This study |
| Δ*rsmA* + pe OX | PAZH13 + pJAK12; St^r^ | This study |
| Δ*rsmA* + p*4570* OX | PAZH13 + pJAK12+ *PA4570* (5117971-5118212) ; St^r^ | This study |
| Δ*rsmA* + p*4570*- *rsmW* OX | PAZH13 + pJAK12+ *PA4570*-*rsmW* ( 5117971-5118537); St^r^ | This study |
| PAO1+ empty-lacZ | lacZ transcriptional fusion attB integration construction plasmid | [[4](#_ENREF_4)] |
| PAO1+*rsmW*S-lacZ | lacZ transcriptional fusion construct; *rsmW* -220→+1::lacZ | This study |
| PAO1+*rsmW*L-lacZ | lacZ transcriptional fusion construct; *rsmW* -1,326→+1::lacZ | This study |
| *rhlR*::IsphoAempty-lacZ | *rhlR*-H04::ISphoA/hah; lacZ transcriptional fusion attB integration construction plasmid; Tc^r^ | This study |
| *rhlR*::IsphoA+*rsmW*S-lacZ | *rhlR*-H04::ISphoA/hah; lacZ transcriptional fusion construct; *rsmW* -220→+1::lacZ; Tc^r^ | This study |
| *rhlR*::IsphoA+*rsmW*L-lacZ | *rhlR*-H04::ISphoA/hah; lacZ transcriptional fusion construct; *rsmW* -1,326→+1::lacZ; Tc^r^ | This study |
| *gacA*::IsphoA+empty-lacZ | *gacA*-E08::ISphoA/hah; lacZ transcriptional fusion attB integration construction plasmid; Tc^r^ | This study |
| *gacA*::IsphoA+*rsmW*S-lacZ | *gacA*-E08::ISphoA/hah; lacZ transcriptional fusion construct; *rsmW* -220→+1::lacZ; Tc^r^ | This study |
| *gacA*::IsphoA+*rsmW*L-lacZ | *gacA*-E08::ISphoA/hah; lacZ transcriptional fusion construct; *rsmW* -1,326→+1::lacZ; Tc^r^ | This study |
| PASK10 | *rsmA*::ΩSm/Spc-lacIQ-P_tac_-*rsmA*; IPTG-inducible, conditional *rsmA* mutant, obtained by allelic exchange | [[2](#_ENREF_2)] |
| PAO1*hfq*^–^ | PAO1 *hfq*::*aadA*; St^r^ | [[5](#_ENREF_5)] |
| Stellar^TM^ | chemical competent cells; F–, endA1, supE44, thi-1, recA1, relA1, gyrA96, phoA, Φ80d lacZΔ M15, Δ (lacZYA - argF) U169, Δ (mrr - hsdRMS - mcrBC), ΔmcrA, λ– | Clontech Laboratories Inc. |
| Stellar^TM^ + pempty | pJAK12; St^r^ | This study |
| Stellar^TM^ + *rsmW* OX | pJAK12-RBS + *rsmW* (5118199-5118345) ; St^r^ | This study |
| Stellar^TM^ + p*4570* OX | pJAK12 + *PA4570* (5117971-5118212) ; St^r^ | This study |
| Stellar^TM^ + p*4570*-*rsmW* OX | pJAK12 + *PA4570*-*rsmW* ( 5117971-5118537); St^r^ | This study |
| **Plasmids** | | |
| pCR2.1 TOPO | cloning vector; Ap^r^, Km^r^ | Invitrogen |
| pΔ*rsmW* Gm | *rsmW* deletion construct (pCR2.1 TOPO backbone); Ap^r^, Km^r^, Gm^r^ | This study |
| p*rsmW* *cis* St | *rsmW* WT “gene-swap” construct (pCR2.1 TOPO backbone); Ap^r^, Km^r^, St^r^ | This study |
| pΔ*PA4570* St | *PA4570* deletion construct (pCR2.1 TOPO backbone); Ap^r^, Km^r^, Gm^r^ | This study |
| p*PA4570* *cis* St | *PA4570* WT “gene-swap” construct (pCR2.1 TOPO backbone); Ap^r^, Km^r^, St^r^ | This study |
| pΔ*PA4570*/ Δ*rsmW* Gm | *PA4570*-*rsmW* deletion construct (pCR2.1 TOPO backbone); Ap^r^, Km^r^, Gm^r^ | This study |
| p*PA4570*/ *rsmW* *cis* St | *PA4570*-*rsmW* *cis*-complementation construct (pCR2.1 TOPO backbone); Ap^r^, Km^r^, St^r^ | This study |
| pJQ200 | broad host range suicide vector, P15A ori, sacB, RP4, gtmR; Gm^r^ | ATCC® 77482™ |
| pCR2.1-P_flgB_-aadA | pCR2.1 cloning vector containing streptomycin resistant cassette, St^r^ | [[6](#_ENREF_6)] |
| pJAK12 | multicopy broad host range overexpression plasmid; St^r^ | ATCC® 77287™ |
| p*rsmW* OX | pJAK12-RBS + *rsmW* (5118199-5118345) ; St^r^ | This study |
| p*4570* OX | pJAK12 + *PA4570* (5117971-5118212) ; St^r^ | This study |
| p*4570*-*rsmW* OX | pJAK12 + *PA4570*-*rsmW*( 5117971-5118537); St^r^ | This study |
| pFLP2 | FLP recombinase expressing plasmid Ap^r^/Cb^r^ | [[7](#_ENREF_7)] |
| mini-CTX-lacZ | lacZ transcriptional fusion attB integration construction plasmid; Tc^r^ | [[4](#_ENREF_4)] |
| *rsmW*S-CTX-lacZ | (5117971-5118191)-*lacZ* transcriptional fusion attB integration construction plasmid; Tc^r^ | This study |
| *rsmW*L-CTX-lacZ | (5116865-5118191)-*lacZ* transcriptional fusion attB integration construction plasmid; Tc^r^ | This study |
| *rsmW*L-CTX-lacZ-Gent | (5116865-5118191)-*lacZ* transcriptional fusion attB integration construction plasmid; Gm^r^ | This study |

a. Antibiotic resistance phenotypes are indicated by r: Ap, ampicillin, Gm gentamicin; Tc, tetracycline; Km, kanamycin; Cb, carbenicillin; St streptomycin

b. Nucleotide numbers correspond to the +1 transcription starts nucleotides.

**REFERENCES**

1. Jacobs MA, Alwood A, Thaipisuttikul I, Spencer D, Haugen E, Ernst S, Will O, Kaul R, Raymond C, Levy R *et al*: **Comprehensive transposon mutant library of *Pseudomonas aeruginosa***. *Proceedings of the National Academy of Sciences of the United States of America* 2003, **100**(24):14339-14344.

2. Kulkarni PR, Jia T, Kuehne SA, Kerkering TM, Morris ER, Searle MS, Heeb S, Rao J, Kulkarni RV: **A sequence-based approach for prediction of CsrA/RsmA targets in bacteria with experimental validation in *Pseudomonas aeruginosa***. *Nucleic acids research* 2014, **42**(11):6811-6825.

3. Pessi G, Williams F, Hindle Z, Heurlier K, Holden MT, Camara M, Haas D, Williams P: **The global posttranscriptional regulator RsmA modulates production of virulence determinants and N-acylhomoserine lactones in *Pseudomonas aeruginosa***. *Journal of bacteriology* 2001, **183**(22):6676-6683.

4. Hoang TT, Kutchma AJ, Becher A, Schweizer HP: **Integration-proficient plasmids for *Pseudomonas aeruginosa*: site-specific integration and use for engineering of reporter and expression strains**. *Plasmid* 2000, **43**(1):59-72.

5. Sonnleitner E, Hagens S, Rosenau F, Wilhelm S, Habel A, Jager KE, Blasi U: **Reduced virulence of a *hfq* mutant of *Pseudomonas aeruginosa O1***. *Microbial pathogenesis* 2003, **35**(5):217-228.

6. Seshu J, Esteve-Gassent MD, Labandeira-Rey M, Kim JH, Trzeciakowski JP, Hook M, Skare JT: **Inactivation of the fibronectin-binding adhesin gene *bbk32* significantly attenuates the infectivity potential of *Borrelia burgdorferi****.* *Molecular microbiology* 2006, **59**(5):1591-1601.

7. Hoang TT, Karkhoff-Schweizer RR, Kutchma AJ, Schweizer HP: **A broad-host-range Flp-FRT recombination system for site-specific excision of chromosomally-located DNA sequences: application for isolation of unmarked *Pseudomonas aeruginosa* mutants**. *Gene* 1998, **212**(1):77-86.
