# Supplementary material for: RsmW, Pseudomonas aeruginosa small non-coding RsmA-binding RNA upregulated in biofilm versus planktonic growth conditions
Source: BMC Microbiol. 2016 Jul 19;16:155. doi: 10.1186/s12866-016-0771-y (PMC4950607; doi:10.1186/s12866-016-0771-y)
Supplement: Additional file 7: Table S3. — Primers used in this study. (DOCX 18 kb) [file 12866_2016_771_MOESM7_ESM.docx]

Table S3. Primers used in this study.

| **Deletion constructs** | |
| --- | --- |
| *rsmW*Inf F1 | AAGCTTCTTCTAGAGGTACCGGTGATCGCCGAGGGCGA |
| *rsmW*Inf R2 | CAGCAGCAGCGAAGTGCTCAGCGCAGGGCGCGG |
| *rsmW*Inf F3 | ACTTCGCTGCTGCTGCCC |
| *rsmW*Inf R4 | ACCGGGACATGAGGACGGCCGCGGCGTTGTGAC |
| *rsmW*Inf F5 | TCCTCATGTCCCGGTCTT |
| *rsmW*InfR6 | TCGATATCGCATGCGGTACCTGCGCAGGCTCAGGGCTT |
| *4570*Inf F1 | GCGAGGAAGCGGAAGGCGACTACATCATCTCCG |
| *4570*Inf R1 | CAGCAGCAGCGAAGTGGCGCTCATCTCCGTGAC |
| *4570*Inf F2 | ACTTCGCTGCTGCTGCCC |
| *4570*Inf R2 | CGTCATCGCAAAGGCTTGTGACAATTTACCGAACAA |
| *4570*Inf F3 | GCCTTTGCGATGACGCCA |
| *4570*Inf R3 | TGCGTATTGGGCGCTCGAGGTAGAACTGCCCGG |
| *4570*-*rsmW*Inf F1 | GCCAGTGTGCTGgaattcGCGACTACATCATCTCCGCCGG |
| *4570*-*rsmW*Inf R2 | CAGCAGCAGCGAAGTGGCGCTCATCTCCGTGACAAGAC |
| *4570*-*rsmW*Inf F3 | ACTTCGCTGCTGCTGCCC |
| *4570*-*rsmW*Inf R4 | GGGACATGAGGATTGTGACAATTTACCGAACAA |
| *4570*-*rsmW*Inf F5 | TCCTCATGTCCCGGTCTTTCCGCAAC |
| *4570*-*rsmW*Inf R6 | GGATATCTGCAgaattcGGGACTTCAGGTAGCTGGCGATG |
| **WT “gene-swap” constructs for *PA4570* and *RsmW*** | |
| *4570*-*rsmW* compl Inf F1 | GCCAGTGTGCTGgaattcGCGACTACATCATCTCCGCCGG |
| *4570*-*rsmW* compl Inf R2 | GTGGTGAATGTGGAATCCCTATCGCGGCGAGGGGCTG |
| *4570*-*rsmW* compl Inf F3 | CACATTCACCACCCTGAATTGAC |
| *4570*-*rsmW* compl Inf R4 | CTTCCCTCATGAATTCTGTTTCCTGTGTGAAATTGTTATC |
| *4570*-*rsmW* compl Inf F5 | ATGAGGGAAGCGGTGATCGC |
| *4570*-*rsmW* compl Inf R6 | GGGACATGAGGATTGTGACAATTTACCGAACAA |
| *4570*-*rsmW* compl Inf F7 | TCCTCATGTCCCGGTCTTTCCGCAAC |
| *4570*-*rsmW* compl Inf R8 | GGATATCTGCAgaattcGGGACTTCAGGTAGCTGGCGATG |
| **p*rsmW* Transcriptional Fusion** | |
| P*rsmW*LfusF1_KpnI | ACGCggtaccATGCAACCCCAGGCCTTCTACCGCGTG |
| P*rsmW*LfusR1_PstI | ATATctgcagCGCAGGGCGCGGGCGTAGCAGAAG |
| P*rsmW*SfusF1_SalI | ACGCgtcgacATGACTTATTTGATCGATGCCTGG |
| P*rsmW*SfusR1_EcoRI | ATATgaattcCGCAGGGCGCGGGCGTAGCAGAAG |
| **Generate CTX-lacZ-Gent** | |
| pucGMF | ACGCgccggcACTTCGCTGCTGCTGCCCAAGGTTG |
| pucGMR | ACGCgccggcACTTCGCTGCTGCTGCCCAAGGTTG |
| **5' RLM-RACE primers** | |
| 5RACEOuter | GCTGATGGCGATGAATGAACACTG |
| 5RACEInner | CGCGGATCCGAACACTGCGTTTGCTGGCTTTGATG |
| 5' RACE Adapter | GCUGAUGGCGAUGAAUGAACACUGCGUUUGCUGGCUUUGAUGAAA |
| Inner 1 | GCCTTTGCCTCCGTTCGTGCGATC |
| Inner 2 | ACTCCGGGAAGGTCGTCCGTGA |
| ***rsmW* northern probe primers** | |
| NB1,*rsmW*senseF | atcgatcgat TAA TAC GAC TCA CTA TAG GG agaCTGAGCCTTTGCGATGACGCCAGG |
| *rsmW*senseR | TTTTTTTTTTTTTTTTTTTTTTTTTTTTTTGCGGAAAGACCGGGACATGAGGAAAG |
| NB1,*rsmW*antisenseF | atcgatcgat TAA TAC GAC TCA CTA TAG GG aga GCGGAAAGACCGGGACATGAGGAAAG |
| *rsmW*antisenseR | TTTTTTTTTTTTTTTTTTTTTTTTTTTTTTCTGAGCCTTTGCGATGACGCC |
| **Removal of ptac promoter** | |
| jak12SDMF_EcorI | TATTCTGAAATGAGCTGTgaattcTGACAATTAATCATCGGC |
| jak12SDMR_EcorI | GCCGATGATTAATTGTCAgaattcACAGCTCATTTCAGAATA |
| ***rsmW* OE RBS minus ptac** | |
| *rsmW*tacF+5118199 | ATATgaattcGAAATGAGCT*GTTGACAATTAATCATCGGCTCGTATAATGTGTGG*TTTGCGATGACGCCAGGACGCGTCATCC |
| *rsmW*SalR 5118345 | ACGCgtcgacGCGGAAAGACCGGGACATGAGGAAAG |
| jktacdel*4570rsmW*R | GCAGGTCGACtctagaTCTAGATTCCTCATCCTTAGGCGTAGGCC |
| **OX primers** |  |
| *PA4570* alone F | ACGCgagctcATGACTTATTTGATCGATGCCTGGC |
| *PA4570* alone R | ATATtctagaAGGCGTCATCGAAAGGCTCAGCGC |
| *PA4570*+*rsmW*F | ACGCgagctcATGACTTATTTGATCGATGCCTGGC |
| *PA4570*+*rsmW*R | GCAGGTCGACtctagaTCTAGATTCCTCATCCTTAGGCGTAGGCC |
| **qRT-PCR primers** | |
| qFabDF | GGTCCAGAATGGTCCTGAAGAG |
| qFabDR | CGATCGAAACCGTAAGGATGGC |
| qrsmZF | GTACAGGGAACACGCAACCC |
| qrsmZR | CTCTTCAGTCCCTCGTCATCATC |
| q*rsmW*F | CTGAGCCTTTGCGATGACGC |
| q*rsmW*R | GCCTTTGCCTCCGTTCGTGCGATC |
| q*PA4570*F | ACTTATTTGATCGATGCCTGGCT |
| q*PA4570*R | CAGAAGAGGAACAGGTTGCGTA |
| q16SF | CAAAACTACTGAGCTAGAGTACG |
| q16SR | TAACATCTCAAGGATCCCAACGGCT |
| qRsmAF | GAGAGACCCTGATGGTAGGTGA |
| qRsmAR | GAGAGACCCTGATGGTAGGTGA |
| qprrF1F | CACTCAACTGGTCGCGAGATC |
| qprrF1R | GCCTGATGAGGAGATAATCTGAAGA |
| qRhlRF | GTTGCATGATCGAGTTGCTGAC |
| qRhlRF2 | CTTCTTCTGGATGTTCTTGTGGTG |
| qLasAF | ATGGACCAGATCCAGGTGAG |
| qLasAR | TAGAGCAGCGAGAAATGCAG |
| qLasBF | AAGCCATCACCGAAGTCAAG |
| qLasBR | CGGATCACCAGTTCCACTTT |
| qPA0531F | TTCATCTGGAACGAGCAGGCTAT |
| qPA0531R | AGGTAATCGAGCAACAGGAACAG |
| qPA2080F | CACATCGATCAACCTGTTCAAGG |
| qPA2080R | GATGTACAGATCAGTGGGGAAGTT |
| qPA3523F | CATGTCAACTACAAGACCGGCTA |
| qPA3523R | CTTTCCACCAATTCGATGAACAGAT |

a. Lowercase letters indicate restriction enzyme sites
